# The comprehensive effects of high-sensitivity C-reactive protein and triglyceride glucose index on cardiometabolic multimorbidity

**DOI:** 10.3389/fendo.2025.1511319

**Published:** 2025-04-01

**Authors:** Bingen Wan, Silin Wang, Sheng Hu, Weiqing Han, Shengyu Qiu, Lingxiao Zhu, Liancheng Ruan, Yiping Wei, Jianjun Xu

**Affiliations:** Department of Thoracic Surgery, The Second Affiliated Hospital, Jiangxi Medical College, Nanchang University, Nanchang, China

**Keywords:** cardiometabolic multimorbidity (CMM), triglyceride glucose index (TyG index), insulin resistance (IR), inflammation, high-sensitivity C-reactive protein (hsCRP)

## Abstract

**Background:**

The triglyceride-glucose index (TyG index) is one of the surrogate markers of insulin resistance, and high-sensitivity C-reactive protein (hsCRP) reflects systemic inflammation. Existing studies suggest that insulin resistance or systemic inflammation may be indicative of cardiometabolic disease, but few of the existing studies have combined the TyG index and inflammation levels before assessing cardiometabolic multimorbidity. Our study data came from the China Health and Retirement Longitudinal Study (CHARLS). Participants in this data were followed for 9 years, and we used these data to conduct a long-term analysis to assess the combined effects of the TyG index and hsCRP on cardiometabolic multimorbidity in Chinese adults over 45 years of age.

**Purpose:**

To study the combined effect of TyG index and hsCRP on cardiometabolic multimorbidity in middle-aged as well as elderly Chinese.

**Method:**

The study data came from the China Health and Retirement Longitudinal Study (CHARLS), which included a total of 4,483 middle-aged and elderly participants who did not have cardiovascular metabolic diseases at baseline, which was from CHARLS 2011, and the last survey was in 2020. A total of five cardiometabolic diseases were considered in this study: diabetes, hypertension, hyperlipidemia, heart disease and stroke. A person was defined as having cardiometabolic multimorbidity when he/she had two or more cardiometabolic diseases at the same time. TyG index (median as cut-off) and hsCRP (1mg/L as cut-off) were each divided into two groups and combined into four groups (Group L-L: TyG index<median & hsCRP<1mg/L; Group H-L: TyG index>=median & hsCRP<1mg/L; Group L-H: TyG index<median & hsCRP>=1mg/L; Group H-H: TyG index>=median & hsCRP>=1mg/L). Multiple regression equations were fitted to analyse the combined influence of TyG index and hsCRP on cardiometabolic multimorbidity.

**Results:**

TyG index increases the risk of CMM events independently of hsCRP, as does the reverse. When the TyG index is elevated and hsCRP is also elevated, this condition significantly increases the danger of cardiometabolic multimorbidity in middle-aged and elderly Chinese.

**Conclusion:**

High levels of TyG index and hsCRP can enhance the danger of cardiometabolic multimorbidity in Chinese middle-aged and elderly people, and the joint use of hsCRP and TyG index assessment may be a better way to achieve primary prevention of cardiometabolic multimorbidity in Chinese middle-aged and elderly people.

## Introduction

Cardiometabolic diseases (CMD), such as stroke, diabetes and hypertension, are becoming increasingly common and represent a major burden on the health of the population, particularly adults over the age of 45 (1). Cardiometabolic multimorbidity (CMM), which is defined as the simultaneous presence of two or more CMDs (including stroke, diabetes mellitus, heart disease, dyslipidemia, and hypertension). In recent years, CMM has received much attention among the population, especially those aged 45 and above (2–5). With the accelerating ageing of the population, cardiovascular health problems are becoming more prevalent in adults over the age of 45, and CMM not only increases the healthcare burden, but also seriously affects people’s quality of life (6, 7).

HsCRP is an inflammatory marker and its elevation is often closely observed in the development of several chronic diseases (8–12). Studies have shown that hsCRP not only has a significant impact on the development of atherosclerosis, but is also a predictor of cardiovascular events (13, 14). Meanwhile, the TyG index, an emerging metabolic index, has shown good potential for use in the early detection of metabolic syndrome, diabetes and cardiovascular disease (15–20). The TyG index is closely linked to lipid metabolism and insulin resistance, and has therefore attracted much attention in the assessment of cardiovascular and metabolic risk (21–24).

The existing research mainly focused on the independent effects of TyG index and hsCRP on cardiovascular risk, but there is relatively limited research on their combined effects and their combined impact on cardiovascular risk in adults older than 45 yearsold. Therefore, by conducting in-depth analyses of the Chinese population aged 45 years or older, we expect to provide valuable guidance for clinical and public health practice to address the growing cardiovascular-metabolic health challenges.

Based on the China Health and Aging National Tracking Survey (CHARLS), this study aims to systematically evaluate the joint effects of TyG index and hsCRP on CMM among Chinese middle-aged and elderly people, and to provide theoretical support for early detection of cardiovascular diseases and appropriate preventive measures.

## Materials and methods

### Data sources

The data used in this study came from the China Longitudinal Study of Health and Retirement (CHARLS), which is free and open to the public(http://charls.pku.edu.cn/). The first official CHARLS survey began in 2011, using a probability proportional to size sampling technique, and included a total of 17,708 individuals from approximately 10,000 households in China. Four surveys were conducted after the baseline survey and the results published (2013, 2015, 2018, and 2020) (25).

### Study population

**Figure 1:**
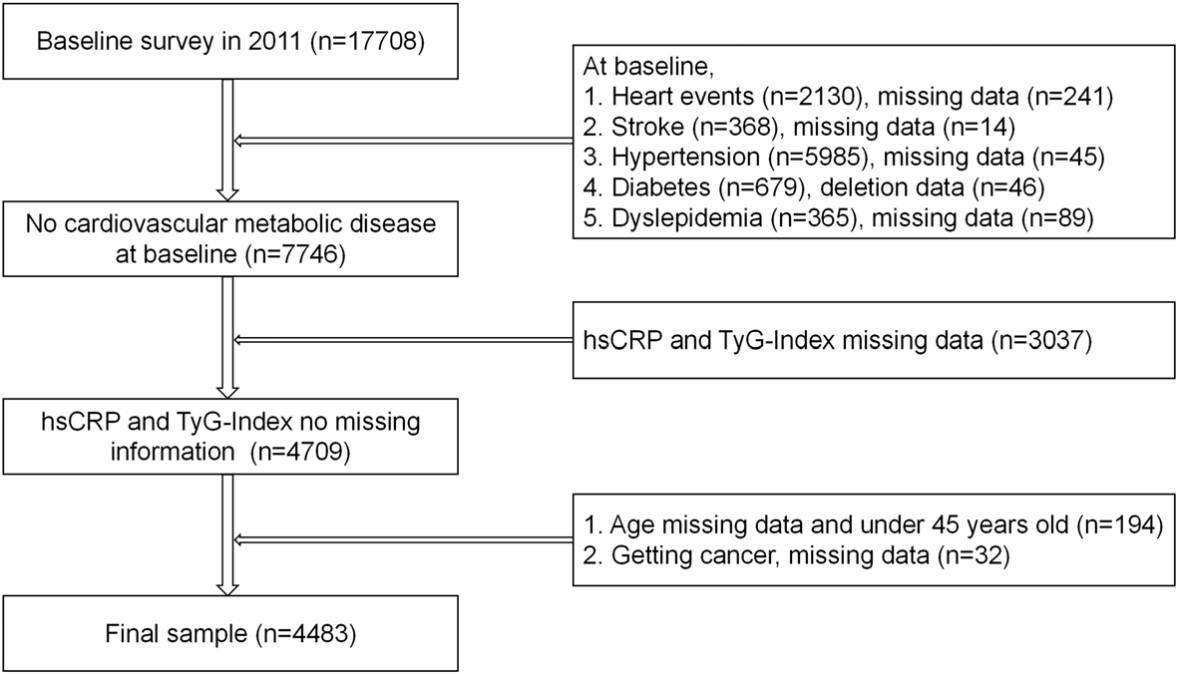
Researcher inclusion flowchart.

Each survey was conducted face-to-face by trained staff and participants using a standardized questionnaire (26). The study used participants from the first visit (2011-2012) as the baseline, with a total of four subsequent follow-up visits. Participants with cardiovascular-metabolic diseases at baseline and missing variables such as sex and age were not included. In the end, our study included 4,483 participants. (see **Figure 1** for details).

### CMM

Cardiovascular metabolic diseases (CMDs) in this study mainly consisted of diabetes mellitus, hypertension, dyslipidemia, stroke and heart disease, and a CMM event was considered to have occurred when 2 or more CMDs occurred (27, 28). Determination of the occurrence of CMD in patients was confirmed by trained volunteers who were interviewed using a standardized questionnaire. Standardized blood pressure measurements were taken (3 measurements with a sphygmomanometer (Omron HEM-7200 monitor) in a relaxed and comfortable environment after at least 5 minutes of rest), and patients were considered to be hypertensive when their systolic blood pressure was ≥140 mmHg and/or diastolic blood pressure was ≥90 mmHg (26, 29); Participants also underwent blood tests to determine whether they had diabetes mellitus or dyslipidemia.

The time when the participant had a second CMD was considered as the time of onset of CMM. Interviewers underwent rigorous training before participating in the questionnaire, and the questionnaire was highly consistent with its international counterparts. The quality of this questionnaire was thus assured.

### Covariates

Gender, age, matrimonial, education, place of living, smoking, drinking, liver disease, kidney disease, sleep time, hsCRP, tyg index, RMS, uric acid, creatinine, uric acid, hemoglobin were the covariates in this study. matrimonial was categorized as ‘Married’ and ‘Other’; Educational level was categorized as ‘Primary school below’, ‘Primary school’, ‘Middle school’, ‘High school and above’; place of living was categorized as ‘Municipalities’, ‘Countryside’; Smoking was categorized as ‘Yes’ and ‘No’; drinking was categorized as ‘Never’, ‘Former’, ‘Current’ and ‘Always’; C-reactive protein (CRP) was quantified by immunoturbidimetric technique. The hsCRP was classified as “<1mg/L” and “>=1mg/L” according to previous literature; TyG index was classified as ‘<median’ and ‘>=median’ based on median (30); RMS was calculated as ASM = 0.193 * weight (kg) + 0.107 * height (cm) - 4.157 * Sex - 0.037 *Age (years) - 2.631, with sex set to 1 for males and 2 for females (31).

### Statistical analysis

Continuous data are presented as mean ± standard deviation (Mean+SD) and categorical data as frequencies and percentages (N (%)). TyG index (median as cut-off) and hsCRP (1mg/L as cut-off) were divided into two groups each and combined into four groups (Group L-L: TyG index<median & hsCRP<1mg/L; Group H-L: TyG index>=median & hsCRP<1mg/L; Group L-H: TyG index<median & hsCRP>=1mg/L; Group H-H: TyG index>=median & hsCRP>=1mg/L). Hazard ratios (HR) and 95% confidence intervals (95% CI) between different TyG index, hsCRP and cardiometabolic multimorbidity were calculated using multiple regression analysis. Group L-L was used as the reference group in the multivariate regression analyses, and model I was only controlled for Gender and Age; model II was controlled for all variables: gender; age; marry; education; residence; drink; smoke; liver disease; kidney disease. Sleeping time; RMS; Blood Urea Nitrogen; Creatinine; Total Cholesterol; Uric Acid; Hemoglobin.

Empower Stats (4.2), SPSS (IBM Statistics 26.0) as well as GraphPad Prism (9.0.0) were performed to analyses the data and statistical differences were considered to exist when the p-value was lower than 0.05.

## Result

17,708 participants took the first survey. After exclusion, a total of 4,483 participants took part in the current study, and these participants took part in at least one of the CHARLS surveys 2 to 5 (see **Figure 1** for details).

In **Table 1**, the mean age of the participants who took part in the first (CHARLS 2011) survey was 57.2 ± 8.9 years, with 2234 (49.8%) males and 2249 (50.2%) females, the majority of the participants were married, with educational attainment concentrated below primary school level, and with a majority of their residence being in rural areas.

**Table 1:** Characteristics of 4483 participants according to TyG-Index and hsCRP levels.

Characteristics	Total	TyG-Index < median & hsCRP <1mg/L	TyG-Index > = median & hsCRP <1mg/L	TyG-Index<median & hsCRP>=1mg/L	TyG-Index>=median & hsCRP>=1mg/L	P
Participants	4483	1616	885	1148	834	
Age	57.2 ± 8.9	56.6 ± 8.9	56.2 ± 8.0	58.7 ± 9.4	57.6 ± 8.8	<0.001
Gender						<0.001
Female	2249 (50.2%)	829 (51.3%)	482 (54.5%)	492 (42.9%)	446 (53.5%)	
Male	2234 (49.8%)	787 (48.7%)	403 (45.5%)	656 (57.1%)	388 (46.5%)	
Marital status						0.198
Married	4080 (91.0%)	1487 (92.0%)	808 (91.3%)	1030 (89.7%)	755 (90.5%)	
Other	403 (9.0%)	129 (8.0%)	77 (8.7%)	118 (10.3%)	79 (9.5%)	
Education						0.606
Primary school below	2041 (45.6%)	717 (44.4%)	419 (47.4%)	527 (45.9%)	378 (45.4%)	
Primary school	933 (20.8%)	347 (21.5%)	178 (20.1%)	246 (21.4%)	162 (19.4%)	
Middle school	979 (21.9%)	358 (22.2%)	175 (19.8%)	253 (22.1%)	193 (23.2%)	
High school and above	526 (11.7%)	193 (12.0%)	112 (12.7%)	121 (10.5%)	100 (12.0%)	
Residence						<0.001
Municipalities	1484 (33.1%)	496 (30.7%)	287 (32.4%)	375 (32.7%)	326 (39.1%)	
Countryside	2999 (66.9%)	1120 (69.3%)	598 (67.6%)	773 (67.3%)	508 (60.9%)	
Drinking						0.365
Never	2591 (57.9%)	929 (57.6%)	522 (59.0%)	643 (56.1%)	497 (59.7%)	
Former	291 (6.5%)	102 (6.3%)	57 (6.4%)	84 (7.3%)	48 (5.8%)	
Current	122 (2.7%)	48 (3.0%)	30 (3.4%)	30 (2.6%)	14 (1.7%)	
Always	1472 (32.9%)	533 (33.1%)	275 (31.1%)	390 (34.0%)	274 (32.9%)	
Smoking						<0.001
No	2645 (59.1%)	992 (61.5%)	542 (61.3%)	611 (53.2%)	500 (60.0%)	
Yes	1834 (40.9%)	622 (38.5%)	342 (38.7%)	537 (46.8%)	333 (40.0%)	
Liver Diseases						0.485
No	4351 (97.1%)	1573 (97.4%)	863 (97.6%)	1110 (96.8%)	805 (96.6%)	
Yes	128 (2.9%)	42 (2.6%)	21 (2.4%)	37 (3.2%)	28 (3.4%)	
Kidney Disease						0.674
No	4277 (95.6%)	1541 (95.7%)	851 (96.3%)	1092 (95.2%)	793 (95.3%)	
Yes	197 (4.4%)	70 (4.3%)	33 (3.7%)	55 (4.8%)	39 (4.7%)	
Sleeping time	6.4 ± 1.9	6.5 ± 1.8	6.4 ± 1.9	6.4 ± 1.9	6.4 ± 1.8	0.228
Relative Muscular Strength (RMS)	1.6 ± 1.6	1.7 ± 1.9	1.6 ± 1.3	1.5 ± 1.8	1.6 ± 1.1	<0.001
Blood Urea Nitrogen (mg/dl)	15.8 ± 4.7	15.9 ± 4.6	15.3 ± 4.1	16.3 ± 5.4	15.3 ± 4.3	<0.001
Creatinine (mg/dl)	0.8 ± 0.2	0.8 ± 0.2	0.8 ± 0.2	0.8 ± 0.3	0.8 ± 0.2	<0.001
Total Cholesterol (mg/dl)	188.4 ± 36.3	184.4 ± 33.0	195.5 ± 36.5	180.9 ± 34.5	199.0 ± 40.5	<0.001
Uric Acid(mg/dl)	4.3 ± 1.2	4.1 ± 1.1	4.3 ± 1.2	4.4 ± 1.2	4.7 ± 1.3	<0.001
Hemoglobin (g/dl)	14.3 ± 2.2	14.1 ± 2.2	14.3 ± 2.1	14.2 ± 2.2	14.5 ± 2.2	<0.001

Results in table: Mean + SD/N (%). P-value*: obtained by Kruskal Wallis rank sum test for continuous variables, and Fisher’s exact probability test for count variables with theoretical number < 10.

A total of 965 (22%) of the 4483 participants included in the study had cardiometabolic multimorbidity at the end of the five surveys conducted by CHARLS, 667 (69%) of the participants had heart disease, 241 (5%) of the participants had stroke, 1802 (40%) of the participants had hypertension, 479 (11%) of the participants had diabetes mellitus and 839 (19%) of the participants had dyslipidemia.

**Figure 2:**
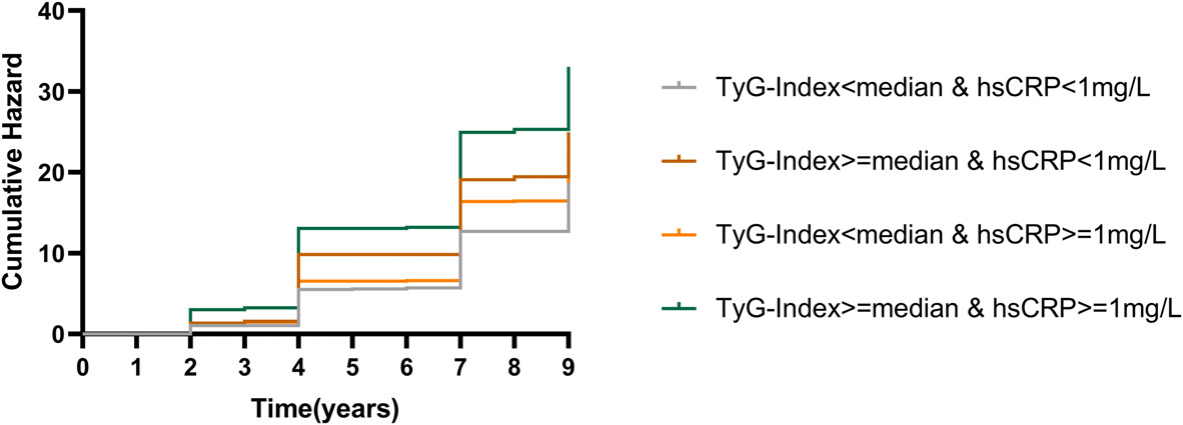
K-M plots of cardiometabolic multimorbidity at different TyG index and hsCRP levels. TyG, triglyceride-glucose index; hsCRP, high-sensitivity C-reactive protein.

The incidence of CMM was 20.0/1000 years for participants in Group L-L; 28.9/1000 years for participants in Group H-L; and 28.9/1000 years for participants in Group L-H had a CMM incidence rate of 24.1/1000 years; and participants in Group H-H had a CMM incidence rate of 37.7/1000 years. **Figure 2** illustrates the KM curves of different combinations of TyG index and hsCRP levels with the cumulative incidence of cardiometabolic multimorbidity.

**Table 2** displays the combined effect of TyG index and hsCRP levels on the hazard of cardiometabolic multimorbidity, using Group L-L as the reference group, and adjusting for confounders (Model II) the group with elevated TyG index alone (HR=1.3, 95%CI=1.1-1.7, p=0.009), the group with elevated hsCRP alone (HR=1.2, 95%CI=1.0-1.5, p=0.036) and the group with both elevated TyG index and hsCRP (HR=2.1, 95%CI=1.7-2.5, p<0.001) participants had a significantly higher risk of CMM. In model II, stroke, hypertension, diabetes and dyslipidemia were all associated with a significantly increased risk of developing CMM with both TyG index and hsCRP elevated compared to the group with neither TyG index nor hsCRP elevated, and although p>0.05 was found for cardiac events, the 95 confidence intervals of the HRs were all greater than 1 (HR=1.3, 95%CI=1.0-1.6, p=0.001, p<0.001). -1.6, p=0.09).

**Table 2:** Risk of cardiometabolic multimorbidity after co-exposure stratified by TyG-index and hsCRP.

	Model I		Model II	
	HR (95% CI)	p	HR (95% CI)	p
CMM (cases/person-years)				
Group L-L (226/11283)	1		1	
Group H-L (277/9883)	1.5 (1.2, 1.8)	<0.001	1.3 (1.1, 1.7)	0.009
Group L-H (188/7805)	1.2 (1.0, 1.5)	0.050	1.2 (1.0, 1.5)	0.036
Group H-H (324/8599)	2.1 (1.8, 2.6)	<0.001	2.1 (1.7, 2.5)	<0.001
Heart (cases/person-years)				
Group L-L (169/11283)	1		1	
Group H-L (185/9883)	1.3 (1.0, 1.6)	0.049	1.3 (1.0, 1.6)	0.054
Group L-H (133/7805)	1.2 (0.9, 1.5)	0.155	1.2 (0.9, 1.5)	0.150
Group H-H (180/8599)	1.3 (1.0, 1.6)	0.048	1.3 (1.0, 1.6)	0.090
Storke (cases/person-years)				
Group L-L (52/11283)	1		1	
Group H-L (54/9883)	1.3 (0.9, 1.9)	0.205	1.3 (0.9, 2.0)	0.231
Group L-H (55/7805)	1.5 (1.1, 2.1)	0.024	1.5 (1.1, 2.2)	0.021
Group H-H (80/8599)	2.0 (1.4, 2.9)	<0.001	1.9 (1.3, 2.8)	0.002
Hypertensive (cases/person-years)				
Group L-L (450/11283)	1		1	
Group H-L (501/9883)	1.3 (1.1, 1.5)	0.002	1.2 (1.0, 1.5)	0.020
Group L-H (353/7805)	1.0 (0.9, 1.2)	0.631	1.0 (0.9, 1.2)	0.862
Group H-H (498/8599)	1.7 (1.4, 2.0)	<0.001	1.7 (1.4, 2.0)	<0.001
Diabetes (cases/person-years)				
Group L-L (105/11283)	1		1	
Group H-L (138/9883)	1.7 (1.3, 2.2)	<0.001	1.6 (1.2, 2.1)	0.002
Group L-H (74/7805)	1.1 (0.8, 1.4)	0.578	1.1 (0.8, 1.4)	0.689
Group H-H (162/8599)	2.3 (1.7, 2.9)	<0.001	2.2 (1.7, 2.9)	<0.001
Dyslipidemia (cases/person-years)				
Group L-L (194/11283)	1		1	
Group H-L (250/9883)	1.5 (1.2, 1.8)	<0.001	1.3 (1.0, 1.6)	0.025
Group L-H (138/7805)	1.0 (0.8, 1.3)	0.721	1.1 (0.9, 1.3)	0.519
Group H-H (257/8599)	2.0 (1.6, 2.4)	<0.001	1.8 (1.4, 2.2)	<0.001

CMM, Cardiometabolic Multimorbidity; HR, hazard ratio; CI, confidence interval; Group L-L, TyG-index<median & hsCRP<1 mg/L; Group H-L, TyG-index>=median & hsCRP<1 mg/L; Group L-H, TyG-index<median & hsCRP>=1 mg/L; Group H-H, TyG-index>=median & hsCRP>=1 mg/L.

**Figure 3:**
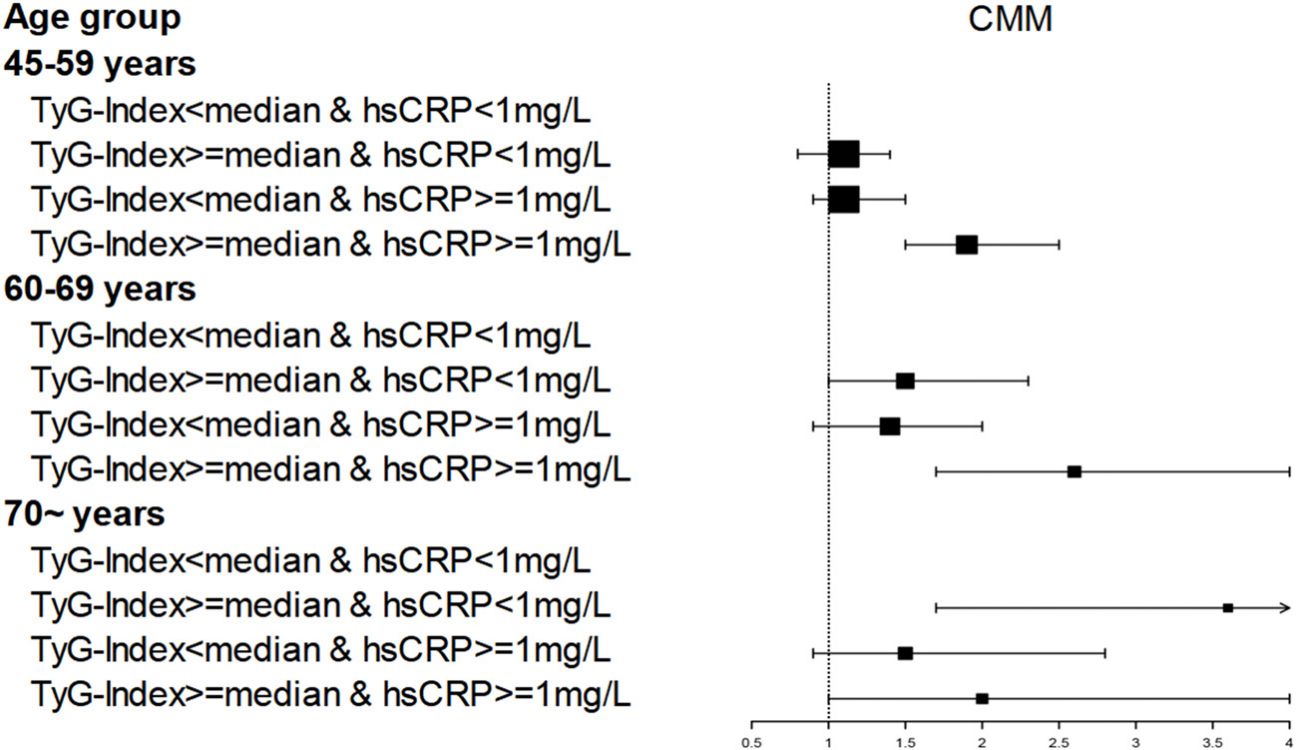
Age affects the association of TyG index and hsCRP levels with CMM occurrence.CMM, Cardiometabolic Multimorbidity; TyG-index, triglyceride-glucose index; hsCRP, high-sensitivity C-reactive protein; HR hazard ratio, CI confidence interval. Number of participants: 45–59 years (n=2903); 60–69 years (n=1090); 70 ~ years (n=490). Dots and lines represent the HR and 95% CI.

As can be seen from **Table 3**, participants had a markedly increased hazard of CMM events when hsCRP was elevated, regardless of the level of TyG index; conversely, participants also had a markedly higher hazard of CMM events when TyG index was elevated, regardless of the level of hsCRP. In age strata, the influence of concurrently elevated levels of TyG index and hsCRP on participants was concentrated in those aged 45-69 years, as shown in **Figure 3** and **Supplementary Figure S1**.

**Table 3:** Reclassification of cardiometabolic multimorbidity risk based on TyG-index and hsCRP.

	CMM		Heart		Storke		Hypertensive		Diabetes		Dyslipidemia	
	HR (95Cl)	P	HR (95Cl)	P	HR (95Cl)	P	HR (95Cl)	P	HR (95Cl)	P	HR (95Cl)	P
Scenario 1 (N of participants)												
TyG-index<median (N=2241)												
hsCRP<1 mg/L	1		1		1		1		1		1	
hsCRP>=1 mg/L	1.3 (1.0, 1.6)	0.044	1.1 (0.8, 1.4)	0.472	1.6 (1.0, 2.4)	0.033	1.1 (0.9, 1.3)	0.328	1.0 (0.7, 1.5)	0.823	1.1 (0.9, 1.5)	0.344
TyG-index>=median (N=2242)												
hsCRP<1 mg/L	1		1		1		1		1		1	
hsCRP>=1 mg/L	1.5 (1.2, 1.8)	<0.001	1.1 (0.8, 1.4)	0.648	1.5 (1.0, 2.1)	0.051	1.2 (1.0, 1.4)	0.106	1.4 (1.1, 1.8)	0.018	1.3 (1.0, 1.6)	0.026
Scenario 1 (N of participants)												
hsCRP<1 mg/L (N=2501)												
TyG-index<median	1		1		1		1		1		1	
TyG-index>=median	1.4 (1.1, 1.7)	0.006	1.3 (1.0, 1.6)	0.054	1.2 (0.8, 1.9)	0.311	1.4 (1.1, 1.6)	<0.001	1.4 (1.1, 1.9)	0.013	1.4 (1.1, 1.7)	0.007
hsCRP>=1 mg/L (N=1982)												
TyG-index<median	1		1		1		1		1		1	
TyG-index>=median	1.6 (1.3, 2.0)	<0.001	1.2 (0.9, 1.6)	0.187	1.2 (0.8, 1.8)	0.325	1.5 (1.2, 1.8)	<0.001	2.0 (1.4, 2.8)	<0.001	1.5 (1.2, 2.0)	0.001

CMM, Cardiometabolic Multimorbidity; HR, hazard ratio; CI, confidence interval.

Model I model adjust for: Gender; Age. Model II model adjust for: Gender; Age; marry; Education; Residence; Drink; Smoking; Liver Diseases; Kidney Disease; Sleeping time; RMS; Blood Urea Nitrogen; Creatinine; Total Cholesterol; Uric Acid; Hemoglobin.

Scenario 1: Effect of hsCRP on cardiometabolic co-morbidities between different TyG-index groups; Scenario 2: Effect of TyG-index on cardiometabolic co-morbidities between different hsCRP groups.

## Discussion

By analysing the health records of 4,483 middle-aged and older Chinese adults followed for 9 years, we found that increased levels of the TyG index and hsCRP led to an increased risk of cardiometabolic multimorbidity, which was most significant in people aged 45-69 years. Furthermore, we found that both TyG index and hsCRP can influence cardiometabolic multimorbidity independently of each other. This result held true after we adjusted for numerous risk factors.

Previous studies have generally assessed the effect of TyG index or hsCRP on cardiometabolic multimorbidity (CMM).

The TyG index may serve as a useful alternative marker of insulin resistance, which has been shown to contribute to atherosclerosis and cardiovascular disease (32–35). Insulin resistance can lead to increased activity of the sympathetic nervous system, making sodium less easily excreted from the body as well as causing vascular excitation (36). Numerous studies have also shown that TyG index is strongly associated with cardiovascular and metabolic diseases (37–39), and Zhao S et al. found that elevated TyG index could lead to arterial stiffness and microvascular damage in northern Shanghai, China (40), and that vascular stiffness and damage play a key role in stroke and cardiac events (41–43). Moreover, several studies have found that the TyG index is strongly associated with the development of cardiovascular metabolic disease, and that elevated TyG index significantly increases the incidence of cardiovascular metabolic diseases (35, 36, 44–48).

hsCRP is one of the more common inflammatory markers, and has been studied by many scholars because of its close relationship with cardiovascular metabolic diseases. The role of inflammation in cardiovascular metabolic diseases is well established, and hsCRP is a well-recognised marker of cardiovascular risk (49, 50). Numerous studies have shown that inflammation levels are significantly associated with atherosclerosis (51, 52). Inflammation has a major role in affecting lipid metabolism and increasing insulin resistance (53–56). Verma et al. found that vascular inflammation is the core factor contributing to atherosclerosis (57), and Ridker et al. demonstrated that not only cholesterol, but also inflammation is not to be underestimated in atherosclerosis risk. Therefore, we should pay attention to the effect of inflammation on atherosclerosis to detect vascular lesions earlier. Many scholars have used the combination of hsCRP and TyG index for the evaluation of cardiovascular and cerebrovascular diseases as well as for the assessment of cancer prognosis (57–60), which shows that the combination of hsCRP and TyG index is of great clinical significance.

Our present study focused on analyzing the joint influence of hsCRP and TyG index on cardiometabolic diseases. We found that participants with both high levels of hsCRP and high levels of TyG index had a significantly higher risk of cardiometabolic diseases than those with low levels of both hsCRP nor TyG index, and this phenomenon is evident in the 45-69 age group. Zuliani et al. have also suggested that indicators of chronic inflammation and insulin resistance should be used more comprehensively when assessing for older adults (61).

We also found that hsCRP significantly assessed the risk of cardiometabolic events when influenced by the TyG index; similarly, the TyG index significantly assessed the risk of cardiometabolic events when influenced by hsCRP, suggesting that hsCRP and TyG indices alone can be used for the early detection of cardiometabolic events in the clinical setting.

The effect of hsCRP and Tyg index on the risk of cardiometabolic multimorbidity may arise from several causes. The mechanisms linking insulin resistance and chronic inflammation to vascular sclerosis, vascular injury, and dyslipidemia are complex. Insulin resistance leads to abnormal lipid metabolism and microvascular injury leading to metabolic diseases and cardiovascular events (33, 40, 62). Under normal circumstances, the endothelial cells of human blood vessels protect the vascular system by synthesizing nitric oxide, while insulin resistance often triggers oxidative stress reactions, leading to increased inflammation and decreased endothelial nitric oxide synthase activity, resulting in reduced production of protective nitric oxide (63–67). In addition, insulin resistance is related to the increase of free fatty acid and reactive oxygen species levels and the onset of dyslipidemia, which will lead to oxidative stress and the release of proinflammatory cytokines, damage insulin signal transduction, and accelerate atherosclerosis (33, 65, 68–70). Furthermore, from a pathological point of view, insulin resistance inhibits PI-3 kinase activity and eNOS expression, ultimately leading to vascular endothelial dysfunction (71, 72). Inflammatory mediators released in response to inflammation can influence insulin regulation of nitric oxide and endothelin-1 affecting vasoregulation, and hsCRP can bind lipoproteins for pro-inflammatory complement activation (73, 74). Inflammation also promotes insulin resistance through the oxidative stress, sympathetic activity and immune responses (16, 41, 75). Insulin resistance can promote the occurrence and development of inflammation, and conversely, the presence of inflammation can also promote insulin resistance.

Sodium dependent glucose transporters 2 inhibitors(SGLT2i)and Glucagon like peptide-1 receptor agonist (GLP-1RA) are both new hypoglycemic drugs. Initially, these two drugs were used to treat diabetes. But with further research, researchers have found that SGLT2i and GLP-1RA also have functions of inhibiting fibrosis, inhibiting cell apoptosis, reducing inflammation, and alleviating oxidative stress (76, 77). And multiple studies have shown that SGLT2i and GLP-1RA have protective effects on the cardiovascular system (78–81). In the near future, SGLT2i and GLP-1RA may have significant implications for CMM.

### Limitations of the study

The CHAARLS database used in this study has a long follow-up period (2011-2020), is rigorous in data collection, and has data covering 450 village-level units in China. However, there are some limitations. First, cardiac events and strokes were obtained by questionnaire, and although the database has a detailed and rigorous process and face-to-face interviews by professionally trained volunteers, there may still be bias from respondents. Second, although we included many possible influencing factors, some unmeasured confounders (e.g., psychiatric scores and economic situation) may still have an impact on the results. Third, since the participants were all from the Chinese region, it may not be representative for regions such as Europe and the United States.

## Conclusion

High levels of TyG index and hsCRP may raise the danger of cardiometabolic multimorbidity in Chinese middle-aged and elderly people, and the joint use of TyG index and hsCRP may be a better way to achieve primary prevention of cardiometabolic multimorbidity in Chinese middle-aged and elderly people.

## Data Availability

The datasets presented in this study can be found in online repositories. The names of the repository/repositories and accession number(s) can be found below: https://charls.charlsdata.com/users/sign_up/agreement/zh-cn.html.

## Supplementary material

Supplementary Figure 1 TyG index and hsCRP are associated with the risk of cardiometabolic multimorbidity when stratified by age, related to **Figure 3**.
