# Supplementary figures and images for: The comprehensive effects of high-sensitivity C-reactive protein and triglyceride glucose index on cardiometabolic multimorbidity
Source: Front Endocrinol (Lausanne). 2025 Apr 1;16:1511319. doi: 10.3389/fendo.2025.1511319 (PMC11996647; doi:10.3389/fendo.2025.1511319)

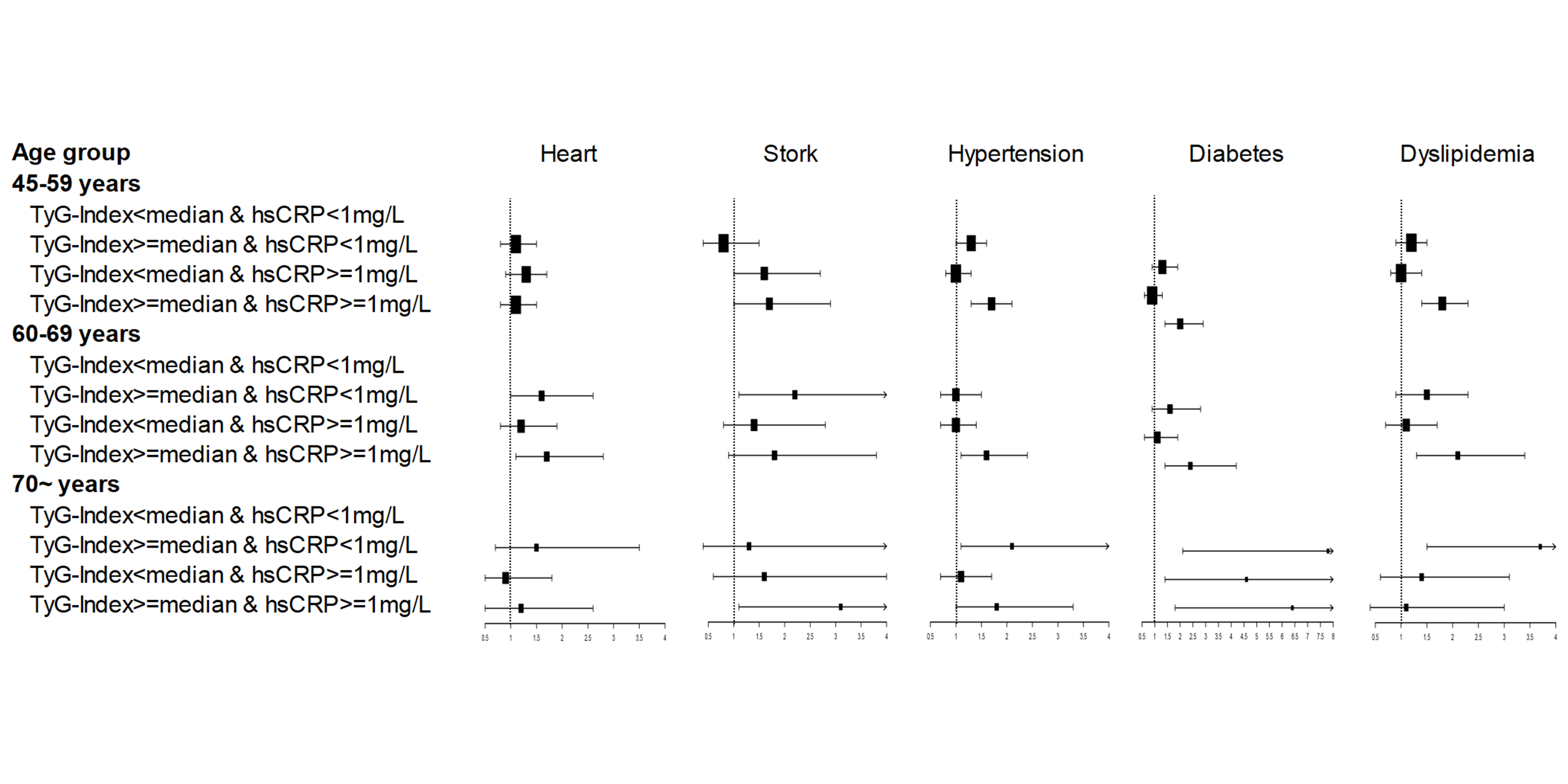

Supplement: Supplementary Figure 1 — TyG index and hsCRP are associated with the risk of cardiometabolic multimorbidity when stratified by age, related to Figure 3 . [file Image1.tif]
