# Supplementary material for: The comprehensive effects of high-sensitivity C-reactive protein and triglyceride glucose index on cardiometabolic multimorbidity
Source: Front Endocrinol (Lausanne). 2025 Apr 1;16:1511319. doi: 10.3389/fendo.2025.1511319 (PMC11996647; doi:10.3389/fendo.2025.1511319)
Supplement: Supplementary file 3 [file DataSheet2.docx]

# Table 1

## Created by EmpowerStats @ Mon, 30 Sep 24 15:37:11 +0800##

#******************** Regarding ALL Following R Code ********************

#*** COPYRIGHT (c) 2010, 2021 X&Y Solutions, ALL RIGHT RESERVED ***********

#******************* www.EmpowerStats.com *********************************

#**************************************************************************

Sys.setlocale(category = 'LC_ALL', locale = 'English_United States.1252');

.libPaths(file.path(R.home(),'library'));

library(doBy);

options(timeout=600);

library(plotrix);

library(stringi);

library(stringr);

library(survival);

library(rms);

library(nnet);

library(car);

library(mgcv);

pdfwd<-6; pdfht<-6;

load('C:/EmpowerRCH/Analysis/cmdcrptyg/202409230.Rdata');

if (length(which(ls()=='EmpowerStatsR'))==0) EmpowerStatsR<-get(ls()[1]);

names(EmpowerStatsR)<-toupper(names(EmpowerStatsR));

originalVNAME<-names(EmpowerStatsR);

ofname<-'cmdcrptyg_1_tbl';

recodevar <- function (var,oldcode,newcode) {

tmp.v <- var

nc.tmp <- length(oldcode)

str <- 0;

if (!is.numeric(oldcode)) {

if (sum(grepl('[|(|)|]|^|*|+|$|.|\\|/', oldcode))==0) {

if (sum(duplicated(substr(oldcode,1,min(nchar(oldcode)))))==0) {oldcode<-paste("^",oldcode,sep=""); str <- 1;}

}

}

for (i in (1:nc.tmp)) {

if (!is.na(oldcode[i]) & oldcode[i] != "NA") {

if (str == 1) {tmp.v[str_detect(var, str_escape('(0,19]'))] <- newcode[i];

} else {tmp.v[var == oldcode[i]]<-newcode[i]; }

} else if (!is.na(newcode[i])) {tmp.v[is.na(var)] <- newcode[i] }

}

if (is.factor(tmp.v)) {tmp.v1<-as.numeric(as.character(tmp.v))} else {tmp.v1<-as.numeric(tmp.v)}

rm(tmp.v); return(tmp.v1)

}

attach(EmpowerStatsR);

sink(paste(ofname,'_datastep.lst',sep=''));

print('Creating new variable: SMOKE.RCD');

SMOKE.RCD<- recodevar(SMOKE,c(0,1,2,3,NA),c(0,1,1,1,NA));

summary(SMOKE.RCD);

EmpowerStatsR<-cbind(EmpowerStatsR,SMOKE.RCD);

rm(SMOKE.RCD);

sink();

vname<-c(NA,'GENDER','GENDER.0','GENDER.1','AGE','MARRY','MARRY.0','MARRY.1','EDU','EDU.1','EDU.2','EDU.3','EDU.4','RURAL','RURAL.0','RURAL.1','DRINK','DRINK.0','DRINK.1','DRINK.2','DRINK.3','SMOKE','SMOKE.0','SMOKE.1','SMOKE.2','SMOKE.3','LIVERE','LIVERE.0','LIVERE.1','KIDNEYE','KIDNEYE.0','KIDNEYE.1','SLEEP','CRP.TYG','CRP.TYG.1','CRP.TYG.2','CRP.TYG.3','CRP.TYG.4','RMS','BL_BUN','BL_CREA','BL_CHO','BL_UA','BL_HGB','SMOKE.RCD','SMOKE.RCD.0','SMOKE.RCD.1')[-1];

vlabel<-c(NA,'Gender',' female',' male','Age','marry',' other',' married','Education',' Primary school below',' Primary school',' middle school',' High school and above','residence',' municipalities',' countryside','DRINK',' 0',' 1',' 2',' 3','SMOKE',' 0',' 1',' 2',' 3','Liver Diseases',' no',' yes','Kidney Disease',' no',' yes','Sleeping time','CRP.TYG',' 1',' 2',' 3',' 4','RMS','Blood Urea Nitrogen (BUN) (mg/dl)urea nitrogen','Creatinine (mg/dl)','Total Cholesterol (mg/dl) Total Cholesterol','Uric Acid(mg/dl)','Hemoglobin (g/dl)','SMOKE recoded',' 0',' 1')[-1];

varused4this <- c('GENDER','AGE','MARRY','EDU','RURAL','DRINK','SMOKE','LIVERE','KIDNEYE','SLEEP','CRP.TYG','RMS','BL_BUN','BL_CREA','BL_CHO','BL_UA','BL_HGB','SMOKE.RCD');

pkgs<-c('gdata');

for (g in pkgs) {

if (!(g %in% rownames(installed.packages()))) install.packages(g,repos='https://cloud.r-project.org');

}

library(gdata);

WD <- EmpowerStatsR; rm(EmpowerStatsR); gc();

title<-'研究人群描述';

wd.subset='';

svy<- 0;

weights.var<- NA;

xvname<-c('GENDER','AGE','MARRY','EDU','RURAL','DRINK','SMOKE.RCD','LIVERE','KIDNEYE','SLEEP','RMS','BL_BUN','BL_CREA','BL_CHO','BL_UA','BL_HGB');

xlv<-c(2,0,2,4,2,4,2,2,2,0,0,0,0,0,0,0);

bvar<-'CRP.TYG'; blv<- 4;

colvname<- NA;

prn<-1;

dec<-1;

##R package## gdata ##R package##;

pvformat<-function(p,dec) {

pp <- sprintf(paste("%.",dec,"f",sep=""),as.numeric(p))

if (is.matrix(p)) {pp<-matrix(pp, nrow=nrow(p)); colnames(pp)<-colnames(p);rownames(pp)<-rownames(p);}

lw <- paste("<",substr("0.00000000000",1,dec+1),"1",sep="");

pp[as.numeric(p)<(1/10^dec)]<-lw

return(pp)

}

numfmt<-function(p,dec) {

if (is.list(p)) p<-as.matrix(p)

pp <- sprintf(paste("%.",dec,"f",sep=""),as.numeric(p))

if (is.matrix(p)) {pp<-matrix(pp, nrow=nrow(p));colnames(pp)<-colnames(p);rownames(pp)<-rownames(p);}

pp[as.numeric(p)>10000000]<- "inf."

pp[is.na(p) | gsub(" ","",p)==""]<- ""

pp[p=="-Inf"]<-"-Inf"

pp[p=="Inf"]<-"Inf"

return(pp)

}

mat2htmltable<-function(mat) {

t1<- apply(mat,1,function(z) paste(z,collapse="</td><td>"))

t2<- paste("<tr><td>",t1,"</td></tr>")

return(paste(t2,collapse=" "))

}

average<-function(mx) {return(mean(mx,na.rm=TRUE))}

mxsum<-function(mx) {return(sum(mx,na.rm=TRUE)) }

stdev<-function(mx) {return(sd(mx,na.rm=TRUE))}

stderr<-function(mx) {return(std.error(mx,na.rm=TRUE))}

mxmedian<-function(mx) {return(median(mx,na.rm=TRUE))}

mxmax<-function(mx) {return(ifelse(sum(!is.na(mx))==0,NA,max(mx,na.rm=TRUE)))}

mxmin<-function(mx) {return(ifelse(sum(!is.na(mx))==0,NA,min(mx,na.rm=TRUE)))}

mxq1<-function(mx) {return(quantile(mx,probs=0.25,na.rm=TRUE))}

mxq3<-function(mx) {return(quantile(mx,probs=0.75,na.rm=TRUE))}

mxp5<-function(mx) {return(quantile(mx,probs=0.05,na.rm=TRUE))}

mxp95<-function(mx) {return(quantile(mx,probs=0.05,na.rm=TRUE))}

mxp90<-function(mx) {return(quantile(mx,probs=0.05,na.rm=TRUE))}

mxp10<-function(mx) {return(quantile(mx,probs=0.05,na.rm=TRUE))}

mx.n<-function(mx) {return(sum(!is.na(mx)))}

gm.n<-function(mx) {return(length(mx[mx>0]))}

gm.mean<-function(mx) {return(exp(average(log(mx[mx>0]))))}

gm.low<-function(mx) {mm<-average(log(mx[mx>0])); ss<-stderr(log(mx[mx>0])); return(exp(mm-1.96*ss))}

gm.upp<-function(mx) {mm<-average(log(mx[mx>0])); ss<-stderr(log(mx[mx>0])); return(exp(mm+1.96*ss))}

matchbyrowname<-function(mx1,mx2,putColumn1,readColumn2) {

tmp.1=rownames(mx1)

if (is.matrix(mx2)) {tmp.2=rownames(mx2)} else {tmp.2=names(mx2)}

for (i in (1:nrow(mx1))) {

tmp.r<-which(tmp.2==tmp.1[i])

if (length(tmp.r)>0) {

if (is.matrix(mx2)) {mx1[i,putColumn1]<-mx2[tmp.r,readColumn2]} else {mx1[i,putColumn1]<-mx2[tmp.r]}

}

}

return(mx1)

}

varfreqpercent<-function(var) {

a<-table(var)

b<-matrix(paste(a, " (", numfmt(a/sum(a)*100,dec), "%)",sep=""),ncol=1)

rownames(b)<-levels(factor(var))

return(b)

}

varmeanstd<-function(var) {

if ((max(var,na.rm=TRUE)==1 | max(var,na.rm=TRUE)==0) & min(var,na.rm=TRUE)==0) {

return(numfmt(average(var)*100,dec))

} else {

return(paste(numfmt(average(var),dec), " + ", numfmt(stdev(var),dec),sep=""))

}

}

v_univariate<-function(x,group,dec,opt,vname,gname) {

vnum <-tapply(x,factor(group),function(mx) sum(!is.na(mx)))

vmean<-numfmt(tapply(x,factor(group),function(mx) mean(mx,na.rm=TRUE)),dec)

vstd <-numfmt(tapply(x,factor(group),function(mx) sd(mx,na.rm=TRUE)),dec)

vmedian <-numfmt(tapply(x,factor(group),function(mx) median(mx,na.rm=TRUE)),dec)

vmin <-numfmt(tapply(x,factor(group),function(mx) ifelse(sum(!is.na(mx))==0,NA,min(mx,na.rm=TRUE))),dec)

vq1 <-numfmt(tapply(x,factor(group),function(mx) quantile(mx,probs=0.25,na.rm=TRUE)),dec)

vq3 <-numfmt(tapply(x,factor(group),function(mx) quantile(mx,probs=0.75,na.rm=TRUE)),dec)

vmax <-numfmt(tapply(x,factor(group),function(mx) ifelse(sum(!is.na(mx))==0,NA,max(mx,na.rm=TRUE))),dec)

tmp<-table(group)

if ((length(tmp)>1) & (min(tmp)>1)) {

pvalue<-try(summary(aov(x~factor(group)))[[1]]$"Pr(>F)"[1])

if (substr(pvalue[1],1,5)=="Error") {

pvalue1<-" ";

} else {

pvalue1<-ifelse(pvalue<0.001,"P < 0.001", paste("P =",pvformat(pvalue,3)))

}

pvalue.npr<-try(kruskal.test(x~factor(group))$p.value)

if (substr(pvalue.npr[1],1,5)=="Error") {

pvalue.npr1<-" ";

} else {

pvalue.npr1<-ifelse(pvalue.npr<0.001, "<0.001",pvformat(pvalue.npr,3))

}

} else {

pvalue1=" "

pvalue.npr1=" "

}

if (opt< 8) o1<-cbind(vnum,vmean,vstd,vmedian,vmin,vq1,vq3,vmax)

if (opt==8) {

vq1.q3<-paste(vq1,"-",vq3)

vrange<-paste(vmin,"-",vmax)

o1<-rbind(vnum,vmean,vstd,vmedian,vq1.q3,vrange)

pvalue<-c(" ",pvalue1,rep(" ",4))

o1<-cbind(o1,pvalue)

rm(vnum,vmean,vstd,vmedian,vmin,vmax,vq1,vq3,pvalue,pvalue1,vq1.q3,vrange)

o1<-rbind(rep(" ",ncol(o1)),o1)

rownames(o1)<-c(vname," N"," Mean"," SD"," Median"," Q1-Q3"," Min-Max")

return(list(o1,pvalue.npr1))

} else {

if (opt==1) o2<-paste(o1[,2], " ± ", o1[,3],sep="")

if (opt==2) o2<-paste(o1[,2], " (", o1[,3], ") ", o1[,4], " ", o1[,6], "-", o1[,7], sep="")

if (opt==3) o2<-paste(o1[,2], " (", o1[,3], ") ", o1[,4], " ", o1[,5], "-", o1[,8], sep="")

if (opt==4) o2<-paste("(",o1[,1],") ",o1[,2], " ± ", o1[,3],sep="")

if (opt==5) o2<-paste("(",o1[,1],") ",o1[,2], " (", o1[,3], ") ", o1[,4], " ", o1[,6], "-", o1[,7], sep="")

if (opt==6) o2<-paste("(",o1[,1],") ",o1[,2], " (", o1[,3], ") ", o1[,4], " ", o1[,5], "-", o1[,8], sep="")

if (opt==7) { oo<-rbind(c(rep(" ",8),pvalue1),cbind(o1," ")) } else {oo<-matrix(o2,ncol=1); rm(o2)}

if (opt<7) {

oo<-rbind(pvalue1,oo)

colnames(oo)<-vname

} else {

colnames(oo)<-c("N","Mean","SD","Median","Min","Q1","Q3","Max","P value")

}

rownames(oo)<-c(gname,paste(" ",names(vnum)))

rm(o1,vnum,vmean,vstd,vmedian,vmin,vmax,vq1,vq3,pvalue,pvalue1)

return(oo)

}

}

rowmatchbind<-function(mx1,mx2,fix9) {

if (fix9) {

p1<-mx1[,ncol(mx1)]; p2<-mx2[,ncol(mx2)]

t1<-t(mx1[,-ncol(mx1)]); t2<-t(mx2[,-ncol(mx2)])

tt<-t(merge(t1,t2,by="row.names",all=TRUE))

tt=cbind(tt[-1,],c(p1,p2))

} else {

t1<-t(mx1);t2<-t(mx2)

tt<-t(merge(t1,t2,by="row.names",all=TRUE))

tt=tt[-1,]

}

rownames(tt)=c(rownames(mx1),rownames(mx2))

return(tt)

}

t1_meanlist<-function(xname,gname,dec,WD1) {

nc<-length(xname); mx<-WD1[,xname];if (nc==1) mx<-as.matrix(mx,ncol=nc)

group<-WD1[,gname]; glv<-levels(factor(group))

xlabel<-vlabelV[match(xname,vnameV)];

xlabel[is.na(xlabel)]<-xname[is.na(xlabel)]

for (i in (1:nc)) {

rlist<-v_univariate(mx[,i],group,dec,8,xlabel[i],"")

r1<-rlist[[1]]

pv.npr1<-as.matrix(rlist[[2]],nrow=1)

rownames(pv.npr1)<-xlabel[i]

if (i==1) {

rr<-r1;pv.npr<-pv.npr1

} else {

rr<-rowmatchbind(rr,r1,TRUE);pv.npr<-rbind(pv.npr,pv.npr1)

}

rm(r1,pv.npr1)

}

ng<-length(table(group))

if (length(glv)>1) {

gclabel<-vlabelZ[match(paste(gname,".",names(table(group)),sep=""),vnameZ)]

if (ng>1) {colnames(rr)<-c(gclabel,"P value")} else {rr<-rr[,-ncol(rr)];colnames(rr)<-gclabel}

} else {

rr<-as.matrix(rr[,-ncol(rr)],ncol=1)

colnames(rr)="Statistics"

}

return(list(rr,pv.npr))

}

t1_mean0 <- function(xname,dec,opt,WD1) {

nc<-length(xname); mx<-WD1[,xname];if (nc==1) mx<-as.matrix(mx,ncol=nc)

oo<-nrow(mx)

vimean<-numfmt(apply(mx,2,average),dec)

vistd<- numfmt(apply(mx,2,stdev),dec)

vinn<-apply(!is.na(mx),2,sum)

vimedian<-numfmt(apply(mx,2,mxmedian),dec)

vimin<-numfmt(apply(mx,2,mxmin),dec)

vimax<-numfmt(apply(mx,2,mxmax),dec)

viq1<-numfmt(apply(mx,2,mxq1),dec)

viq3<-numfmt(apply(mx,2,mxq3),dec)

gn<-apply(mx,2,gm.n)

gmean<-numfmt(apply(mx,2,gm.mean),dec)

gmeanlow<-numfmt(apply(mx,2,gm.low),dec)

gmeanupp<-numfmt(apply(mx,2,gm.upp),dec)

if (opt==1) {oo<-paste(vimean," ± ",vistd,sep="")}

if (opt==2) {oo<-paste(vimean," (",vistd,") ",vimedian," (",vimin,"-",vimax,")",sep="")}

if (opt==3) {oo<-paste(vimean," (",vistd,") ",vimedian," (",viq1,"-",viq3,")",sep="")}

if (opt==4) {oo<-paste(gmean," (",gmeanlow," ",gmeanupp,")",sep="")}

if (opt==5) {oo<-paste("(",vinn,") ",vimean," ± ",vistd,sep="")}

if (opt==6) {oo<-paste("(",vinn,") ",vimean," (",vistd,") ",vimedian," (",vimin,"-",vimax,")",sep="")}

if (opt==7) {oo<-paste("(",vinn,") ",vimean," (",vistd,") ",vimedian," (",viq1,"-",viq3,")",sep="")}

if (opt==8) {oo<-paste("(",gn,") ",gmean," (",gmeanlow," ",gmeanupp,")",sep="")}

oo<-matrix(oo,nrow=ncol(mx))

tmp<-vlabelV[match(xname,vnameV)]; tmp[is.na(tmp)]<-xname[is.na(tmp)]

rownames(oo)<-tmp

return(oo)

}

t1_mean <- function(xname, gname, dec, opt, WD1) {

nc<-length(xname); mx<-WD1[,xname];if (nc==1) mx<-as.matrix(mx,ncol=nc)

grp<-WD1[,gname]; ngrp<-length(levels(factor(grp)))

oo<-summary(factor(grp)); oocomp<-is.na(cbind(grp,mx))

pp<-" "; st.diff<-" ";

for (i in (1:nc)) {

vimean<-tapply(mx[,i],factor(grp),average)

vistd<-tapply(mx[,i],factor(grp),stdev)

vinn<-table(grp[!is.na(mx[,i])])

vimedian<-numfmt(tapply(mx[,i],factor(grp),mxmedian),dec)

vimin<-numfmt(tapply(mx[,i],factor(grp),mxmin),dec)

vimax<-numfmt(tapply(mx[,i],factor(grp),mxmax),dec)

viq1<-numfmt(tapply(mx[,i],factor(grp),mxq1),dec)

viq3<-numfmt(tapply(mx[,i],factor(grp),mxq3),dec)

gn<-tapply(mx[,i],factor(grp),gm.n)

gmean<-numfmt(tapply(mx[,i],factor(grp),gm.mean),dec)

gmeanlow<-numfmt(tapply(mx[,i],factor(grp),gm.low),dec)

gmeanupp<-numfmt(tapply(mx[,i],factor(grp),gm.upp),dec)

grpcomp<-grp[apply(oocomp[,c(1,i+1)],1,sum)==0]

if (opt==4 | opt==8) {

mxi<-mx[,i]; grpi<-grp

mxi2<-log(mxi[mxi>0]);grpi2<-grpi[mxi>0]

if (length(levels(factor(grpi2)))>1) {

pvalue<-summary(aov(mxi2~factor(grpi2)))[[1]]$"Pr(>F)"[1]

pp1<-ifelse(pvalue<0.001, "<0.001",pvformat(pvalue,3))

} else {pp1<-" "}

} else {

if (length(levels(factor(grpcomp)))>1) {

pvalue<-summary(aov(mx[,i]~factor(grp)))[[1]]$"Pr(>F)"[1]

pp1<-ifelse(pvalue<0.001, "<0.001",pvformat(pvalue,3))

} else {pp1<-" "}

}

if (length(levels(factor(grpcomp)))>1) {

pvalue.npr<-kruskal.test(mx[,i]~factor(grp))$p.value

pp1.npr<-ifelse(pvalue.npr<0.001, "<0.001",pvformat(pvalue.npr,3))

} else {pp1.npr<-" "}

if (ngrp==2) {

stddiff <- abs(vimean[2] - vimean[1])/sqrt((vistd[2]^2 + vistd[1]^2)/2)

se <- sqrt((vinn[1]+vinn[2])/(vinn[1]*vinn[2]) + stddiff^2/(2*(vinn[1]+vinn[2])))

stddiff.l <- stddiff - 1.96 * se

stddiff.u <- stddiff + 1.96 * se

vi.stdiff<-paste(numfmt(stddiff,dec), " (", numfmt(stddiff.l,dec), ", ", numfmt(stddiff.u,dec), ")", sep="")

st.diff<-rbind(st.diff,vi.stdiff)

}

pp<-rbind(pp,cbind(pp1,pp1.npr))

if (opt==1) {ooi<-paste(numfmt(vimean,dec)," ± ",numfmt(vistd,dec),sep="")}

if (opt==2) {ooi<-paste(numfmt(vimean,dec)," (",numfmt(vistd,dec),") ",vimedian," (",vimin,"-",vimax,")",sep="")}

if (opt==3) {ooi<-paste(numfmt(vimean,dec)," (",numfmt(vistd,dec),") ",vimedian," (",viq1,"-",viq3,")",sep="")}

if (opt==4) {ooi<-paste(gmean," (",gmeanlow," ",gmeanupp,")",sep="")}

if (opt==5) {ooi<-paste("(",vinn,") ",numfmt(vimean,dec)," ± ",numfmt(vistd,dec),sep="")}

if (opt==6) {ooi<-paste("(",vinn,") ",numfmt(vimean,dec)," (",numfmt(vistd,dec),") ",vimedian," (",vimin,"-",vimax,")",sep="")}

if (opt==7) {ooi<-paste("(",vinn,") ",numfmt(vimean,dec)," (",numfmt(vistd,dec),") ",vimedian," (",viq1,"-",viq3,")",sep="")}

if (opt==8) {ooi<-paste("(",gn,") ",gmean," (",gmeanlow," ",gmeanupp,")",sep="")}

oo=rbind(oo,ooi)

}

tmp.cname<-vlabelZ[match(paste(gname,".",colnames(oo),sep=""),vnameZ)]

if (ngrp==2) {

oo<-cbind(oo,st.diff,pp)

colnames(oo)<-c(tmp.cname,"Standardize diff.","P value", "P value*")

} else {

oo=cbind(oo,pp)

colnames(oo)<-c(tmp.cname,"P value", "P value*")

}

tmp<-vlabelV[match(xname,vnameV)]; tmp[is.na(tmp)]<-xname[is.na(tmp)]

rownames(oo)<-c("N", tmp)

return(oo)

}

t1_freq0 <- function(xname,WD1) {

nc<-length(xname); mx<-WD1[,xname];if (nc==1) mx<-as.matrix(mx,ncol=nc)

for (i in (1:nc)) {

tt<-table(mx[,i],useNA="no")

ss<-round(tt/sum(tt)*100,dec)

ooi<-paste(format(tt)," (",numfmt(ss,dec),"%)",sep="")

ooi<-matrix(c(" ",ooi),ncol=1)

tmp.rname<-vlabelZ[match(paste(xname[i],".",names(tt),sep=""),vnameZ)]

tmp.rname[is.na(tmp.rname)]<-names(tt)

tmp<-vlabelV[match(xname[i],vnameV)]; if (is.na(tmp)) tmp<-xname[i]

rownames(ooi)<-c(tmp,paste(" ",tmp.rname,sep=""))

ifelse(i==1, oo<-ooi, oo<-rbind(oo,ooi))

}

return(oo)

}

t1_freq <- function(xname, gname, WD1) {

nc<-length(xname); mx<-WD1[,xname];if (nc==1) mx<-as.matrix(mx,ncol=nc)

grp<-WD1[,gname]; ngrp<-length(levels(factor(grp)))

for (i in (1:nc)) {

t1<-table(mx[,i],factor(grp),useNA="no")

pvalue<-chisq.test(t1,correct=FALSE)$p.value

pp1<-ifelse(pvalue<0.001, "<0.001", pvformat(pvalue,3))

ooi<-cbind(matrix(rep(" ",times=ncol(t1)),nrow=1), pp1)

pp1.exact="-"

coltot <- min(apply(t1,2,sum))

rowtot <- min(apply(t1,1,sum))

if (min(c(coltot, rowtot)) < 10) {

if ((coltot*rowtot/sum(t1)<10) & max(dim(t1))<4) {

pexact <- try(fisher.test(t1)$p.value)

if (substr(pexact,1,5)!="Error") pp1.exact<-ifelse(pexact<0.001,"<0.001",pvformat(pexact,3))

}

}

p1<-prop.table(t1,2)

tb1<-matrix(paste(format(t1)," (", numfmt(p1*100,dec), "%)", sep=""),nrow=nrow(t1))

if (ngrp==2) {

t <- p1[-1, 2]; c <- p1[-1, 1]; k <- nrow(p1)-1; r <- k

s <- matrix(rep(0, k * r), ncol = k)

for (ii in 1:k) {

for (j in 1:r) {

if (ii == j) {s[ii, j] <- 0.5 * (t[ii]*(1-t[ii])+c[ii]*(1-c[ii]))

} else {s[ii, j] <- -0.5 * (t[ii]*t[j] + c[ii]*c[j])

}

}

}

e <- rep(1, k); e <- diag(e);

if (!(nrow(s)==1 && ncol(s)==1 && s[1,1]==0)) {

s <- solve(s, e)

tc1 <- t - c

tc2 <- t - c

stddiff <- sqrt(t(tc1) %*% s %*% tc2)

n1 <- sum(t1[,1]); n2 <- sum(t1[,2]); n <- n1+n2

se <- sqrt(1/(n1/n*n2) + stddiff^2/(2*n))

stddiff.l <- stddiff - 1.96 * se

stddiff.u <- stddiff + 1.96 * se

vi.stdiff<-paste(numfmt(stddiff,dec), " (", numfmt(stddiff.l,dec), ", ", numfmt(stddiff.u,dec), ")", sep="")

ooi<-cbind(matrix(rep(" ",times=ncol(t1)),nrow=1), vi.stdiff, pp1, pp1.exact)

tb1<-cbind(tb1,matrix(" ",nrow=nrow(tb1),ncol=3))

} else {

ooi<-cbind(matrix(rep(" ",times=ncol(t1)),nrow=1), "", pp1, pp1.exact)

tb1<-cbind(tb1,matrix(" ",nrow=nrow(tb1),ncol=3))

}

} else {

ooi<-cbind(matrix(rep(" ",times=ncol(t1)),nrow=1), pp1, pp1.exact)

tb1<-cbind(tb1,matrix(" ",nrow=nrow(tb1),ncol=2))

}

tmp<-vlabelV[match(xname[i],vnameV)]; if (is.na(tmp)) tmp<-xname[i]

rownames(ooi)<-tmp

tmp.rname<-vlabelZ[match(paste(xname[i],".",rownames(t1),sep=""),vnameZ)]

tmp.rname[is.na(tmp.rname)]<-rownames(t1)

rownames(tb1)<-paste(" ",tmp.rname,sep="")

ooi=rbind(ooi,tb1)

tmp.cname<-vlabelZ[match(paste(gname,".",colnames(t1),sep=""),vnameZ)]

if (ngrp==2) {colnames(ooi)<-c(tmp.cname, "Standardize diff.", "P value", "P value*")

} else {colnames(ooi)<-c(tmp.cname,"P value", "P value*"); }

ifelse(i==1, oo<-ooi, oo<-rbind(oo,ooi))

}

return(oo)

}

if (!is.na(weights.var)) {weights<-WD[,weights.var];} else {weights<-1;}

WD<-cbind(WD,weights);

vlabelN<-(substr(vlabel,1,1)==" ");

vlabelZ<-vlabel[vlabelN];vlabelV<-vlabel[!vlabelN]

vnameV<-vname[!vlabelN];vnameZ<-vname[vlabelN];

w<-c("<!DOCTYPE html><html lang='zh'><head><meta charset='utf-8'></head><body>")

w<-c(w,paste("<h2>", title, "</h2>"))

allvname<-c(xvname,bvar,colvname); allvname<-allvname[!is.na(allvname)];

WD<-data.frame(WD,TOT_=1)[,c(allvname,"TOT_")];

if (is.na(colvname)) {

nclv<-1; clvb<-"Total"; clvb_<-"Total"

} else {

clv<-levels(factor(WD[,colvname])); nclv<-length(clv)+1

clvb_<-vlabelZ[match(paste(colvname,".",clv,sep=""),vnameZ)];

clvb_[is.na(clvb_)]<-clv[is.na(clvb_)];

clvb<-c(paste(vlabelV[vnameV==colvname],clvb_,sep="="),"Total");

clvb_<-c(clvb_,"Total")

WD<-WD[!is.na(WD[,colvname]),]

}

if (is.na(bvar)) {ncc<-1; tt00<-"";

} else {blv<-levels(factor(WD[,bvar])); ncc<-length(blv);

blvb_<-vlabelZ[match(paste(bvar,".",blv,sep=""),vnameZ)];

blvb_[is.na(blvb_)]<-blv[is.na(blvb_)];

if (ncc==2) {tt00<-c(blvb_,"Standardize diff.","P-value","P-value*")

} else {tt00<-c(blvb_,"P-value","P-value*"); }

WD<-WD[!is.na(WD[,bvar]),];

}

opt<-prn;

if (!is.numeric(opt) | opt<1) opt<-1

prnopt<-c("Mean+SD / N(%)","Mean(SD) Median (Min-Max) / N(%)","Mean(SD) Median (Q1-Q3) / N(%)", "Geometric Mean (95% CI) / N(%)",

"(N) Mean+SD / N(%)","(N) Mean(SD) Median (Min-Max) / N(%)","(N) Mean(SD) Median (Q1-Q3) / N(%)", "(N) Geometric Mean (95% CI) / N(%)",

"List N Mean SD Median Q1-Q3 Min-Max")

xv0<-xvname[xlv==0]; xv1<-xvname[xlv>0]; nxv0<-sum(xlv==0); nxv1<-sum(xlv>0);

stprn<-strsplit(prnopt[opt],"/")[[1]]

for (k in 1:nclv) {

tt<-tt00; ttnp<-c("","")

if (!is.na(colvname) & k<nclv) {WD1<-WD[WD[,colvname]==clv[k],]; } else {WD1<-WD;}

if (!is.na(bvar)) {

if (opt<=8) {

if (nxv0>0) tt<-rbind(tt,t1_mean(xv0,bvar,dec,opt,WD1))

if (nxv1>0) tt<-rbind(tt,t1_freq(xv1,bvar,WD1))

} else {

if (nxv0>0) {

t0.tmp<-t1_meanlist(xv0,bvar,dec,WD1);

tt<-rbind(tt,t0.tmp[[1]])

tvnpr<-t0.tmp[[2]]; tvnpr<-cbind(rownames(tvnpr),tvnpr)

ttnp<-rbind(ttnp,c("","Non-parametric(Kruskal Wallis) test P-value"), tvnpr)

}

if (nxv1>0) {

t1.tmp<-t1_freq(xv1,bvar,WD1);

tt<-rbind(tt,t1.tmp[,-ncol(t1.tmp)])

tvfish<-t1.tmp[,ncol(t1.tmp)]; tvfish<-as.matrix(tvfish[tvfish!=" "],ncol=1)

tvfish<-cbind(rownames(tvfish),tvfish)

ttnp<-rbind(ttnp,c("","Fisher exact test P-value"),tvfish)

}

}

} else {

if (opt<=8) {

if (nxv0>0) tt<-rbind(tt,stprn[1],t1_mean0(xv0,dec,opt,WD1))

if (nxv1>0) tt<-rbind(tt,"N (%)",t1_freq0(xv1,WD1))

} else {

if (nxv0>0) tt<-rbind(tt,"Statistics",t1_meanlist(xv0,"TOT_",dec,WD1)[[1]])

if (nxv1>0) tt<-rbind(tt,"N (%)",t1_freq0(xv1,WD1))

}

}

rname<-rownames(tt); tt<-cbind(rname,tt);

if (!is.na(bvar)) {tt[1,1]<-vlabel[vname==bvar];} else {tt[1,1]<-"";}

if (!is.na(colvname)) w<-c(w,"</br>",clvb[k])

w<-c(w,"</br><table border=3>", mat2htmltable(tt), "</table>")

if (length(ttnp)>2) w<-c(w,"</br><table border=3>", mat2htmltable(ttnp), "</table>")

}

# TABLE 2

## Created by EmpowerStats @ Mon, 30 Sep 24 15:27:03 +0800##

#******************** Regarding ALL Following R Code ********************

#*** COPYRIGHT (c) 2010, 2021 X&Y Solutions, ALL RIGHT RESERVED ***********

#******************* www.EmpowerStats.com *********************************

#**************************************************************************

Sys.setlocale(category = 'LC_ALL', locale = 'English_United States.1252');

.libPaths(file.path(R.home(),'library'));

library(doBy);

options(timeout=600);

library(plotrix);

library(stringi);

library(stringr);

library(survival);

library(rms);

library(nnet);

library(car);

library(mgcv);

pdfwd<-6; pdfht<-6;

load('C:/EmpowerRCH/Analysis/cmdcrptyg/202409230.Rdata');

if (length(which(ls()=='EmpowerStatsR'))==0) EmpowerStatsR<-get(ls()[1]);

names(EmpowerStatsR)<-toupper(names(EmpowerStatsR));

originalVNAME<-names(EmpowerStatsR);

ofname<-'cmdcrptyg_3_tbl';

vname<-c(NA,'GENDER','GENDER.0','GENDER.1','AGE','MARRY','MARRY.0','MARRY.1','EDU','EDU.1','EDU.2','EDU.3','EDU.4','RURAL','RURAL.0','RURAL.1','DRINK','DRINK.0','DRINK.1','DRINK.2','DRINK.3','SMOKE','SMOKE.0','SMOKE.1','SMOKE.2','SMOKE.3','LIVERE','LIVERE.0','LIVERE.1','KIDNEYE','KIDNEYE.0','KIDNEYE.1','SLEEP','CRP.TYG','CRP.TYG.1','CRP.TYG.2','CRP.TYG.3','CRP.TYG.4','RMS','CMD','CMD.0','CMD.1','BL_BUN','BL_CREA','BL_CHO','BL_UA','BL_HGB')[-1];

vlabel<-c(NA,'Gender',' female',' male','Age','marry',' other',' married','Education',' Primary school below',' Primary school',' middle school',' High school and above','residence',' municipalities',' countryside','DRINK',' 0',' 1',' 2',' 3','SMOKE',' 0',' 1',' 2',' 3','Liver Diseases',' no',' yes','Kidney Disease',' no',' yes','Sleeping time','CRP.TYG',' 1',' 2',' 3',' 4','RMS','CMD',' 0',' 1','Blood Urea Nitrogen (BUN) (mg/dl)urea nitrogen','Creatinine (mg/dl)','Total Cholesterol (mg/dl) Total Cholesterol','Uric Acid(mg/dl)','Hemoglobin (g/dl)')[-1];

varused4this <- c('GENDER','AGE','MARRY','EDU','RURAL','DRINK','SMOKE','LIVERE','KIDNEYE','SLEEP','CRP.TYG','RMS','CMD','BL_BUN','BL_CREA','BL_CHO','BL_UA','BL_HGB');

pkgs<-c('gdata','geepack','mgcv');

for (g in pkgs) {

if (!(g %in% rownames(installed.packages()))) install.packages(g,repos='https://cloud.r-project.org');

}

library(gdata);

library(geepack);

library(mgcv);

WD <- EmpowerStatsR; rm(EmpowerStatsR); gc();

title<-'多个回归方程';

wd.subset='';

weights.var<- NA;

yvname<-c('CMD');

ydist<-c('binomial');

ylink<-c('logit');

ylv<-c(2);

avname<-c('GENDER','AGE');

saf<-c(0,0);

alv<-c(2,0);

svname<-c('GENDER','AGE','MARRY','EDU','RURAL','DRINK','SMOKE','LIVERE','KIDNEYE','SLEEP','RMS','BL_BUN','BL_CREA','BL_CHO','BL_UA','BL_HGB');

sdf<-c(0,0,0,0,0,0,0,0,0,0,0,0,0,0,0,0);

slv<-c(2,0,2,4,2,4,4,2,2,0,0,0,0,0,0,0);

prn<-1;

xvname<-c('CRP.TYG');

sxf<-c(0);

xlv<-c(4);

par1<-1;

chk<- 0;

cox<- 0;

timevar<- NA;

vname.start<- NA;

subjvname<- NA;

gee.TYPE<-NA;

bvar<- NA;

colvname<- NA;

par3<-1;

dec<-1;

##R package## gdata geepack mgcv ##R package##;

pvformat<-function(p,dec) {

pp <- sprintf(paste("%.",dec,"f",sep=""),as.numeric(p))

if (is.matrix(p)) {pp<-matrix(pp, nrow=nrow(p)); colnames(pp)<-colnames(p);rownames(pp)<-rownames(p);}

lw <- paste("<",substr("0.00000000000",1,dec+1),"1",sep="");

pp[as.numeric(p)<(1/10^dec)]<-lw

return(pp)

}

numfmt<-function(p,dec) {

if (is.list(p)) p<-as.matrix(p)

pp <- sprintf(paste("%.",dec,"f",sep=""),as.numeric(p))

if (is.matrix(p)) {pp<-matrix(pp, nrow=nrow(p));colnames(pp)<-colnames(p);rownames(pp)<-rownames(p);}

pp[as.numeric(p)>10000000]<- "inf."

pp[is.na(p) | gsub(" ","",p)==""]<- ""

pp[p=="-Inf"]<-"-Inf"

pp[p=="Inf"]<-"Inf"

return(pp)

}

varstats<-function(var,vlvl,dec) {

if (length(vlvl)==1 & vlvl[1]==0) {

return(paste(numfmt(mean(var,na.rm=TRUE),dec),numfmt(sd(var,na.rm=TRUE),dec),sep="+"))

} else {

a<-table(var)

b<-matrix(paste(a, " (", numfmt(a/sum(a)*100,dec), "%)",sep=""),ncol=1)

return(c(" ",b[match(vlvl,names(a))]))

}

}

mat2htmltable<-function(mat) {

t1<- apply(mat,1,function(z) paste(z,collapse="</td><td>"))

t2<- paste("<tr><td>",t1,"</td></tr>")

return(paste(t2,collapse=" "))

}

setgam<-function(fml,yi) {

if (ydist[yi]=="") ydist[yi]<-"gaussian"

if (ydist[yi]=="exact") ydist[yi]<-"binomial"

if (ydist[yi]=="breslow") ydist[yi]<-"binomial"

if (ydist[yi]=="gaussian") mdl<-try(gam(formula(fml),weights=wdtmp$weights,data=wdtmp, family=gaussian(link="identity")))

if (ydist[yi]=="binomial") mdl<-try(gam(formula(fml),weights=wdtmp$weights,data=wdtmp, family=binomial(link="logit")))

if (ydist[yi]=="poisson") mdl<-try(gam(formula(fml),weights=wdtmp$weights,data=wdtmp, family=poisson(link="log")))

if (ydist[yi]=="gamma") mdl<-try(gam(formula(fml),weights=wdtmp$weights,data=wdtmp, family=Gamma(link="inverse")))

if (ydist[yi]=="negbin") mdl<-try(gam(formula(fml),weights=wdtmp$weights,data=wdtmp, family=negbin(c(1,10), link="log")))

return(mdl)

}

setgee<-function(fml,yi) {

if (ydist[yi]=="") ydist[yi]<-"gaussian"

if (ydist[yi]=="exact") ydist[yi]<-"binomial"

if (ydist[yi]=="breslow") ydist[yi]<-"binomial"

if (ydist[yi]=="gaussian") md<-try(geeglm(formula(fml),id=wdtmp[,subjvname],corstr=gee.TYPE,family="gaussian",weights=wdtmp$weights,data=wdtmp))

if (ydist[yi]=="binomial") md<-try(geeglm(formula(fml),id=wdtmp[,subjvname],corstr=gee.TYPE,family="binomial",weights=wdtmp$weights,data=wdtmp))

if (ydist[yi]=="poisson") md<-try(geeglm(formula(fml),id=wdtmp[,subjvname],corstr=gee.TYPE,family="poisson",weights=wdtmp$weights,data=wdtmp))

if (ydist[yi]=="gamma") md<-try(geeglm(formula(fml),id=wdtmp[,subjvname],corstr=gee.TYPE,family="Gamma",weights=wdtmp$weights,data=wdtmp))

if (ydist[yi]=="negbin") md<-try(geeglm.nb(formula(fml),id=wdtmp[,subjvname],corstr=gee.TYPE,weights=wdtmp$weights,data=wdtmp))

return(md)

}

setglm<-function(fml,yi) {

if (ydist[yi]=="") ydist[yi]<-"gaussian"

if (ydist[yi]=="exact") ydist[yi]<-"binomial"

if (ydist[yi]=="breslow") ydist[yi]<-"binomial"

if (ydist[yi]=="gaussian") md<-try(glm(formula(fml),family="gaussian",weights=wdtmp$weights,data=wdtmp))

if (ydist[yi]=="binomial") md<-try(glm(formula(fml),family="binomial",weights=wdtmp$weights,data=wdtmp))

if (ydist[yi]=="poisson") md<-try(glm(formula(fml),family="poisson",weights=wdtmp$weights,data=wdtmp))

if (ydist[yi]=="gamma") md<-try(glm(formula(fml),family="Gamma",weights=wdtmp$weights,data=wdtmp))

if (ydist[yi]=="negbin") md<-try(glm.nb(formula(fml),weights=wdtmp$weights,data=wdtmp))

return(md)

}

mdl2oo<-function(mdl, xxname, opt) {

if (is.na(mdl[[1]][1])) return(list(rep("",times=length(xxname)),""))

if (substr(mdl[[1]][1],1,5)=="Error") return(list(rep("",times=length(xxname)),""))

gs<-summary(mdl); print(mdl$formula); print(gs)

if (opt=="gam") {gsparm <- gs$p.table;tmpn<-gs$n;

} else {gsparm <- gs$coefficients;tmpn <- sum(gs$df[c(1,2)]);}

gsp<-gsparm[match(xxname,rownames(gsparm)),]

if (length(xxname)==1) {beta<-gsp[1]; se<-gsp[2]; pv<-gsp[4];

} else {beta<-gsp[,1]; se<-gsp[,2]; pv<-gsp[,4]; }

ci1<- beta-1.96*se; ci2<- beta+1.96*se

pvx<-substr(rep("****",length(pv)),1,(pv<=0.05)+(pv<=0.01)+(pv<=0.001))

if (colprn==3) {pvv<-pvx;} else {pvv<-pvformat(pv,dec+2);}

if ((colprn!=2) & (gs$family[[2]]=="log" | gs$family[[2]]=="logit")) {

o1<-paste(numfmt(exp(beta),dec)," (",numfmt(exp(ci1),dec),", ",numfmt(exp(ci2),dec),")",sep="")

} else {

if (colprn<3) {o1<-paste(numfmt(beta,dec), " (",numfmt(ci1,dec),", ",numfmt(ci2,dec),")",sep="")

} else {o1<-paste(numfmt(beta,dec), "+",numfmt(se,dec),sep="");}

}

o1<-paste(o1,pvv); o1[is.na(beta)]<-NA

if (length(xxname)>1) {

if (gs$family[[2]]=="log" | gs$family[[2]]=="logit") {

o1[is.na(o1) & substr(xxname,1,7)=="factor("]<-"1.0"

} else {o1[is.na(o1) & substr(xxname,1,7)=="factor("]<-"0";}

o1[is.na(o1)]<-"";

}

return(list(o1,tmpn))

}

recodevar <- function (var,oldcode,newcode) {

tmp.v <- var

nc.tmp <- length(oldcode)

for (i in (1:nc.tmp)) {tmp.v[(var==oldcode[i])]=newcode[i]}

if (is.factor(tmp.v)) {tmp.v1<-as.numeric(as.character(tmp.v))} else {tmp.v1<-as.numeric(tmp.v)}

rm(tmp.v); return(tmp.v1)

}

rankvar <- function(var, num) {

qprobs <- 1/num

if (num>2) {for (i in (2:(num-1))) {qprobs <- c(qprobs, 1/num * i) } }

outvar <- rep(0, times=length(var))

outvar[is.na(var)] <- NA

cutpoints <- quantile(var,probs=qprobs, na.rm=TRUE)

for (k in (1:length(cutpoints))) { outvar[var>=cutpoints[k]] <- k; }

return(outvar)

}

removeNA<-function(i,j,m,wdf) {

vvv<-c(yvname[i],adjvv[[m]],subjvname,colvname,bvar,vname.start,timevar);

if (j<=nx) {vvv<-c(vvv,xvname[j]);} else {vvv<-c(vvv,xvname);}

vvv<-vvv[!is.na(vvv)]; vvv<-vvv[vvv>" "]

tmp<-is.na(wdf[,vvv]);

return(wdf[apply(tmp,1,sum)==0,])

}

if (!is.na(weights.var)) {weights<-WD[,weights.var];} else {weights<-1;}

WD<-cbind(WD,weights);

vlabelN<-(substr(vlabel,1,1)==" ");

vlabelZ<-vlabel[vlabelN];vlabelV<-vlabel[!vlabelN]

vnameV<-vname[!vlabelN];vnameZ<-vname[vlabelN]

w<-c("<!DOCTYPE html><html lang='zh'><head><meta charset='utf-8'></head><body>")

w<-c(w,paste("<h2>", title, "</h2>"))

if (length(avname)>0) {

if (sum((saf=="s" | saf=="S") & alv>0)>0) w<-c(w,"</br>Spline smoothing only applies for continuous variables")

if (!is.na(subjvname) & (sum((saf=="s" | saf=="S") & alv==0)>0)) w<-c(w,"</br>Generalized estimate equation could not be used with spline smoothing terms")

}

if (length(svname)>0) {

if (sum((sdf=="s" | sdf=="S") & slv>0)>0) w<-c(w,"</br>Spline smoothing only applies for continuous variables")

if (!is.na(subjvname) & (sum((sdf=="s" | sdf=="S") & slv==0)>0)) w<-c(w,"</br>Generalized estimate equation could not be used with spline smoothing terms")

}

allvname<-c(yvname,xvname,colvname,bvar,avname,svname,subjvname,vname.start,timevar,"weights");

allvname<-allvname[!is.na(allvname)]

WD<-WD[,allvname];

if (!is.na(subjvname)) WD<-WD[order(WD[,subjvname]),]

sxf<-as.numeric(sxf);sxf[is.na(sxf)]<-0;

if (sum(sxf>1 & xlv>0)>0) w<-c(w,"Categorizing only applies to continuous variables");

if (sum(sxf>1 & xlv==0)>0) {

t.xname<-NA;t.xlv<-NA; nx<-length(xvname)

for (i in 1:nx) {

if (sxf[i]>1 & xlv[i]==0) {

tmp.Xi<- rankvar(WD[,xvname[i]],sxf[i])

tmp.newcode <- tapply(WD[,xvname[i]],tmp.Xi,function(z) median(z,na.rm=TRUE))

tmp.low <- tapply(WD[,xvname[i]],tmp.Xi,function(z) min(z,na.rm=TRUE))

tmp.upp <- tapply(WD[,xvname[i]],tmp.Xi,function(z) max(z,na.rm=TRUE))

tmp.Xi2<- recodevar(tmp.Xi,(1:sxf[i])-1,tmp.newcode)

tmp.Xi<-cbind(tmp.Xi,tmp.Xi2)

tmp.NM<-paste(xvname[i],c("grp","grp.cont"),sep=".")

colnames(tmp.Xi)<-tmp.NM

WD<-cbind(WD,tmp.Xi)

t.xname<-c(t.xname,tmp.NM)

t.xlv<-c(t.xlv,sxf[i],0)

vnameV<-c(vnameV,tmp.NM)

vlabelV<-c(vlabelV,paste(vlabelV[vnameV==xvname[i]],c("group","group trend")))

vnameZ<-c(vnameZ,paste(tmp.NM[1],(1:sxf[i])-1,sep="."))

vlabelZ<-c(vlabelZ,paste(tmp.low,"-",tmp.upp))

} else {

t.xname<-c(t.xname,xvname[i]); t.xlv<-c(t.xlv,xlv[i])

}

}

xvname<-t.xname[-1]; xlv<-t.xlv[-1];

}

if (!is.na(subjvname)) {

if (length(avname)>0) saf<-rep(0,length(saf));

if (length(svname)>0) sdf<-rep(0,length(sdf));

WD<-WD[order(WD[,subjvname]),];

}

ny=length(yvname); nx=length(xvname);

xb<-vlabelV[match(xvname,vnameV)]; xb[is.na(xb)]<-xvname[is.na(xb)]

yb<-vlabelV[match(yvname,vnameV)]; yb[is.na(yb)]<-yvname[is.na(yb)]

xvname_ <- xvname

xvname_[xlv>0]<-paste("factor(",xvname[xlv>0],")",sep="")

xxname_<-list(NA); xxlbl_<-list(NA); xxlvl_<-list(NA)

for (j in (1:nx)) {

if (xlv[j]==0) {

xxname_[[j+1]]<-xvname[j];xxlbl_[[j+1]]<-xb[j];xxlvl_[[j+1]]<-0

} else {

xxlvl_[[j+1]]<-levels(factor(WD[,xvname[j]]))

tmp<-paste(xvname[j],".",xxlvl_[[j+1]],sep="")

xxlbl_[[j+1]]<-c(xb[j],vlabelZ[match(tmp,vnameZ)])

xxlbl_[[j+1]]<-paste(c("",rep("  ",length(xxlbl_[[j+1]])-1)),xxlbl_[[j+1]])

xxname_[[j+1]]<-c(xvname[j],paste("factor(",xvname[j],")",xxlvl_[[j+1]],sep=""))

}

}

xxname_<-xxname_[-1]; xxlbl_<-xxlbl_[-1]; xxlvl_<-xxlvl_[-1];

if (nx==1) par1<-1;

if (nx!=length(svname) & par1==4) par1<-1;

if (is.na(par1) | par1<1) par1<-1;

if (par1>1 & par1 < 4) {

tmp1<-xxname_[[1]]; tmp2<-xxlbl_[[1]]

for (j in 2:nx) {tmp1<-c(tmp1,xxname_[[j]]); tmp2<-c(tmp2,xxlbl_[[j]]);}

xxname_[[nx+1]]<-tmp1; xxlbl_[[nx+1]]<-tmp2;

xvname_<-c(xvname_,paste(xvname_,collapse="+"))

}

contx<-(sum(xlv>0)==0)

if (par1==3 & !is.na(bvar)) {w<-c(w,"</br>Column stratified variable was ignored"); bvar<-NA; bvname<-NA;}

fmlm<-" "; fmlb<-"Non-adjusted"; tmp<-""; adjvv<-list(NA); adjvb<-"None";

fmlm4<-list(rep("",nx))

fmlp<-ifelse(!is.na(subjvname), "gee", "glm");

na=0; avb=""; smoothav<-0; nadjm<-0

if (length(avname)>0) {

na<-length(avname)

avb<-vlabelV[match(avname,vnameV)];

avname_ <- avname

smoothavi<-((saf=="s" | saf=="S") & alv==0)

smoothav<-sum(smoothavi)

smoothavname<-avname[smoothavi]

avname_[smoothavi]<-paste("s(",avname[smoothavi],")",sep="")

avb1<-avb

avb1[smoothavi]<-paste(avb[smoothavi],"(Smooth)",sep="")

avname_[alv>0]<-paste("factor(",avname[alv>0],")",sep="")

fmlm<-c(fmlm,paste("+",paste(avname_,collapse="+")))

fmlb<-c(fmlb,"Adjust")

nadjm<-nadjm+1; tmp<-c(tmp,"I"); adjvv[[nadjm+1]]<-avname;

adjvb<-c(adjvb, paste(avb1, collapse="; "))

fmlp<-c(fmlp,ifelse(!is.na(subjvname), "gee", ifelse(smoothav>0, "gam", "glm")))

fmlm4<-c(fmlm4, list(rep("",nx)))

}

ns=0; svb=""; smoothsv<-0

if (length(svname)>0) {

svb<-vlabelV[match(svname,vnameV)];

svname_ <- svname

smoothsvi<-((sdf=="s" | sdf=="S") & slv==0)

smoothsv<-sum(smoothsvi)

smoothsvname<-svname[smoothsvi]

svname_[smoothsvi]<-paste("s(",svname[smoothsvi],")",sep="")

svb1<-svb

svb1[smoothsvi]<-paste(svb[smoothsvi],"(Smooth)",sep="")

svname_[slv>0]<-paste("factor(",svname[slv>0],")",sep="")

if (par1==4) {

fmlm<-c(fmlm, paste(fmlm[length(fmlm)],"+"));

fmlm4<-c(fmlm4, list(svname_));

} else {

fmlm<-c(fmlm,paste("+",paste(svname_,collapse="+")));

fmlm4<-c(fmlm4, list(rep("",nx)));

}

fmlb<-c(fmlb,"Adjust")

nadjm<-nadjm+1; tmp<-c(tmp,"II"); adjvv[[nadjm+1]]<-svname

adjvb2 <- paste(svb1, collapse="; ")

if (par1 == 4) {

if (avb>"") {adjvb2<-paste(avb1, "AND one in (", adjvb2,") respectively for each exposure X");

} else { adjvb2<-paste("ONE of (", adjvb2,") respectively for each exposure X");}

}

adjvb<-c(adjvb, adjvb2)

fmlp<-c(fmlp,ifelse(!is.na(subjvname), "gee", ifelse(smoothsv>0, "gam", "glm")))

}

if (chk==0 & length(fmlm)>1) {

fmlm<-fmlm[-1]; fmlb<-fmlb[-1]; tmp<-tmp[-1]; adjvv<-adjvv[-1]; adjvb<-adjvb[-1]; fmlp<-fmlp[-1]; fmlm4<-fmlm4[-1];

}

if (nadjm>1) fmlb<-paste(fmlb,tmp)

nmdl<-length(fmlm)

if (is.na(bvar) & !is.na(colvname) & nmdl==1 & par1!=3) {if ((ny==1) | (nx==1 & contx)) {bvar<-colvname; colvname<-NA;}}

if (is.na(colvname)) {

nclv<-1; clvb<-"Total"; clvb_<-"Total"

} else {

clv<-levels(factor(WD[,colvname])); nclv<-length(clv)+1

clvb_<-vlabelZ[match(paste(colvname,".",clv,sep=""),vnameZ)]; clvb_[is.na(clvb_)]<-clv[is.na(clvb_)];

clvb<-c(paste(vlabelV[vnameV==colvname],clvb_,sep="="),"Total");

clvb_<-c(clvb_,"Total")

WD<-WD[!is.na(WD[,colvname]),]

}

if (is.na(bvar)) {

blvb<-"Total"; blvb_<-"Total"

} else {

blv<-levels(factor(WD[,bvar])); nblv<-length(blv)+1

blvb_<-vlabelZ[match(paste(bvar,".",blv,sep=""),vnameZ)]; blvb_[is.na(blvb_)]<-blv[is.na(blvb_)];

blvb<-c(paste(vlabelV[vnameV==bvar],blvb_,sep="="),"Total");

blvb_<-c(blvb_,"Total")

WD<-WD[!is.na(WD[,bvar]),]

}

aa<-c(1,2,3,4)

for (i in 1:4) {

for (j in c(1:4)[-i]) {

for (k in c(1:4)[-c(i,j)]) aa<-rbind(aa,c(i,j,k,c(1:4)[-c(i,j,k)]))

}

}

colprn<-prn;

if (is.na(par3) | par3<1) par3<-1

rord<-aa[par3,]

if (!is.na(bvar)) {prn<-"S";

} else {

if (par3>1) {

rordc<-ifelse(rord[4]==1,3,4)

prn<-c("G","Y","M","X")[rord[rordc]]

if (prn=="X") {

if (!contx & nx>1) prn<-ifelse(nmdl>1, "M", ifelse(ny>nx & contx, "X", "Y"))

if (!contx & nx==1) prn<-"CX"

if (par1==2) {tmp<-ifelse(rordc==4,ifelse(rord[3]==1,2,3),2); prn<-c("G","Y","M","X")[rord[tmp]];}

}

} else {

prn<-ifelse(nmdl>1, "M", ifelse(ny>nx & contx, "X", "Y"))

if (par1==2) prn<-ifelse(nmdl>1, "M", "Y")

}

if (par1==3) prn<-"UM"

}

sink(paste(ofname,".lst",sep=""))

if (par1==2) {xbgn<-nx+1; xend<-nx+1;} else {xbgn<-1; xend<-nx;}

if (prn=="Y") {

tt<-c(0,0,0,0,"Exposure",yb); nn<-c(0,0,0,0,yb);

for (k in (1:nclv)) {

wdtmp0<-WD;

if (!is.na(colvname)) {

if (k<nclv) wdtmp0<-WD[WD[,colvname]==clv[k],];

print(paste("Stratified by",colvname, ":", clvb[k]))

}

for (m in 1:nmdl) {

for (j in (xbgn:xend)) {

colj<-cbind(k,0,m,j,xxlbl_[[j]])

nnj <-c(k,0,m,j)

for (i in (1:ny)) {

if (par1==4) {fml<-paste(yvname[i],"~",xvname_[j],fmlm[m],fmlm4[[m]][j]);} else {fml<-paste(yvname[i],"~",xvname_[j],fmlm[m]);}

wdtmp<-removeNA(i,j,m,wdtmp0)

if (!is.na(colvname)) {if (k==nclv) fml<-paste(fml,"+factor(",colvname,")",sep="");}

if (fmlp[m]=="gam") tmp.mdl<-setgam(fml,i)

if (fmlp[m]=="gee") tmp.mdl<-setgee(fml,i)

if (fmlp[m]=="glm") tmp.mdl<-setglm(fml,i)

tmpooi<-mdl2oo(tmp.mdl,xxname_[[j]],fmlp[m])

colj<-cbind(colj,tmpooi[[1]]); nnj<-c(nnj,tmpooi[[2]])

}

tt<-rbind(tt,colj); nn<-rbind(nn,nnj)

}

}

}

}

if (prn=="S") {

tt<-c(0,0,0,0,"Exposure",blvb); nn<-c(0,0,0,0,blvb);

for (k in (1:nclv)) {

wdtmp0<-WD;

if (!is.na(colvname)) {

if (k<nclv) wdtmp0<-WD[WD[,colvname]==clv[k],];

print(paste("Stratified by",colvname, ":", clvb[k]))

}

for (i in (1:ny)) {

for (m in 1:nmdl) {

for (j in (xbgn:xend)) {

colj<-cbind(k,i,m,j,xxlbl_[[j]]);

nnj <- c(k,i,m,j)

for (b in (1:nblv)) {

print(paste("Stratified by",bvar, ":", blvb[b]))

if (par1==4) {fml<-paste(yvname[i],"~",xvname_[j],fmlm[m],fmlm4[[m]][j]);} else {fml<-paste(yvname[i],"~",xvname_[j],fmlm[m]);}

if (b<nblv) {wdtmp1<-wdtmp0[wdtmp0[,bvar]==blv[b],];} else {wdtmp1<-wdtmp0; fml<-paste(fml,"+factor(",bvar,")",sep="");}

wdtmp<-removeNA(i,j,m,wdtmp1)

if (!is.na(colvname)) {if (k==nclv) fml<-paste(fml,"+factor(",colvname,")",sep="");}

if (fmlp[m]=="gam") tmp.mdl<-setgam(fml,i)

if (fmlp[m]=="gee") tmp.mdl<-setgee(fml,i)

if (fmlp[m]=="glm") tmp.mdl<-setglm(fml,i)

tmpooi<-mdl2oo(tmp.mdl,xxname_[[j]],fmlp[m])

colj<-cbind(colj,tmpooi[[1]]); nnj<-c(nnj,tmpooi[[2]])

}

tt<-rbind(tt,colj); nn<-rbind(nn,nnj)

}

}

}

}

}

if (prn=="M") {

tt<-c(0,0,0,0,"Exposure",fmlb); nn<-c(0,0,0,0,fmlb)

for (k in (1:nclv)) {

wdtmp0<-WD;

if (!is.na(colvname)) {

if (k<nclv) wdtmp0<-WD[WD[,colvname]==clv[k],];

print(paste("Stratified by",colvname, ":", clvb[k]))

}

for (i in 1:ny) {

for (j in xbgn:xend) {

colj<-cbind(k,i,0,j,xxlbl_[[j]]); nnj<-c(k,i,0,j)

for (m in 1:nmdl) {

if (par1==4) {fml<-paste(yvname[i],"~",xvname_[j],fmlm[m],fmlm4[[m]][j]);} else {fml<-paste(yvname[i],"~",xvname_[j],fmlm[m]);}

wdtmp<-removeNA(i,j,m,wdtmp0)

if (!is.na(colvname)) {if (k==nclv) fml<-paste(fml,"+factor(",colvname,")",sep="");}

if (fmlp[m]=="gam") tmp.mdl<-setgam(fml,i)

if (fmlp[m]=="gee") tmp.mdl<-setgee(fml,i)

if (fmlp[m]=="glm") tmp.mdl<-setglm(fml,i)

tmpooi<-mdl2oo(tmp.mdl,xxname_[[j]],fmlp[m])

colj<-cbind(colj,tmpooi[[1]]); nnj<-c(nnj,tmpooi[[2]])

}

tt<-rbind(tt,colj); nn<-rbind(nn,nnj)

}

}

}

}

if (prn=="X") {

tt<-c(0,0,0,0,"Outcome",xb); nn<-c(0,0,0,0,xb);

for (k in (1:nclv)) {

wdtmp0<-WD;

if (!is.na(colvname)) {

if (k<nclv) wdtmp0<-WD[WD[,colvname]==clv[k],];

print(paste("Stratified by",colvname, ":", clvb[k]))

}

for (m in 1:nmdl) {

for (i in (1:ny)) {

colj<-cbind(k,i,m,0,yb[i])

nnj <-c(k,i,m,0)

for (j in (1:nx)) {

if (par1==4) {fml<-paste(yvname[i],"~",xvname_[j],fmlm[m],fmlm4[[m]][j]);} else {fml<-paste(yvname[i],"~",xvname_[j],fmlm[m]);}

wdtmp<-removeNA(i,j,m,wdtmp0)

if (!is.na(colvname)) {if (k==nclv) fml<-paste(fml,"+factor(",colvname,")",sep="");}

if (fmlp[m]=="gam") tmp.mdl<-setgam(fml,i)

if (fmlp[m]=="gee") tmp.mdl<-setgee(fml,i)

if (fmlp[m]=="glm") tmp.mdl<-setglm(fml,i)

tmpooi<-mdl2oo(tmp.mdl,xxname_[[j]],fmlp[m])

colj<-cbind(colj,tmpooi[[1]]); nnj<-c(nnj,tmpooi[[2]])

}

tt<-rbind(tt,colj); nn<-rbind(nn,nnj)

}

}

}

}

if (prn=="CX") {

tt<-c(0,0,0,0,"Outcome",xxlbl_[[1]][-1]); nn<-c(0,0,0,0,xb[1]);

nxl<-length(xxlbl_[[1]])-1

for (k in (1:nclv)) {

wdtmp0<-WD;

if (!is.na(colvname)) {

if (k<nclv) wdtmp0<-WD[WD[,colvname]==clv[k],];

print(paste("Stratified by",colvname, ":", clvb[k]))

}

for (m in 1:nmdl) {

for (i in (1:ny)) {

colj<-c(k,i,m,0,yb[i])

nnj <-c(k,i,m,0)

fml<-paste(yvname[i],"~",xvname_[1],fmlm[m]);

wdtmp<-removeNA(i,1,m,wdtmp0)

if (!is.na(colvname)) {if (k==nclv) fml<-paste(fml,"+factor(",colvname,")",sep="");}

if (fmlp[m]=="gam") tmp.mdl<-setgam(fml,i)

if (fmlp[m]=="gee") tmp.mdl<-setgee(fml,i)

if (fmlp[m]=="glm") tmp.mdl<-setglm(fml,i)

tmpooi<-mdl2oo(tmp.mdl,xxname_[[1]],fmlp[m])

colj<-c(colj,tmpooi[[1]][-1]); nnj<-c(nnj,tmpooi[[2]])

tt<-rbind(tt,colj); nn<-rbind(nn,nnj)

}

}

}

}

if (prn=="UM") {

tt<-c(0,0,0,0,"Exposure","Univariable","Multivariable"); nn<-c(0,0,0,0,"Univariable","Multivariable")

for (k in (1:nclv)) {

wdtmp0<-WD;

if (!is.na(colvname)) {

if (k<nclv) wdtmp0<-WD[WD[,colvname]==clv[k],];

print(paste("Stratified by",colvname, ":", clvb[k]))

}

for (i in 1:ny) {

for (m in 1:nmdl) {

colm<-rep(NA,6); nnm<-rep(NA,5)

for (j in 1:(nx+1)) {

colj<-cbind(k,i,m,j,xxlbl_[[j]]); nnj<-c(k,i,m,j)

if (par1==4) {fml<-paste(yvname[i],"~",xvname_[j],fmlm[m],fmlm4[[m]][j]);} else {fml<-paste(yvname[i],"~",xvname_[j],fmlm[m]);}

wdtmp<-removeNA(i,j,m,wdtmp0)

if (!is.na(colvname)) {if (k==nclv) fml<-paste(fml,"+factor(",colvname,")",sep="");}

if (fmlp[m]=="gam") tmp.mdl<-setgam(fml,i)

if (fmlp[m]=="gee") tmp.mdl<-setgee(fml,i)

if (fmlp[m]=="glm") tmp.mdl<-setglm(fml,i)

tmpooi<-mdl2oo(tmp.mdl,xxname_[[j]],fmlp[m])

colj<-cbind(colj,tmpooi[[1]]); nnj<-c(nnj,tmpooi[[2]])

if (j<=nx) {colm<-rbind(colm,colj); nnm<-rbind(nnm,nnj);

} else {colm<-cbind(colm[-1,],tmpooi[[1]]); nnm<-cbind(nnm[-1,],tmpooi[[2]]);}

}

tt<-rbind(tt,colm); nn<-rbind(nn,nnm)

}

}

}

}

sink()

if (!contx & prn!="CX") rord<-c(rord[rord!=4],4)

if (prn=="X" | prn=="CX") rord<-rord[rord!=4]

if (prn=="Y") rord<-rord[rord!=2]

if (prn=="M") rord<-rord[rord!=3]

if (nx==1 & contx) rord<-rord[rord!=4]

if (nmdl==1) rord<-rord[rord!=3]

if (ny==1) rord<-rord[rord!=2]

if (is.na(colvname)) rord<-rord[rord!=1]

if (length(rord)==0) rord<-1

nrr<-length(rord)

for (i in nrr:1) {nn<-nn[order(as.numeric(nn[,rord[i]])),];tt<-tt[order(as.numeric(tt[,rord[i]])),];}

if (nn[2,4]>0) {nn[,4]<-c("Exposure",xb[as.numeric(nn[-1,4])]); } else {nn<-nn[,-4];}

if (nn[2,3]>0) {nn[,3]<-c("Model",fmlb[as.numeric(nn[-1,3])]); } else {nn<-nn[,-3];}

if (nn[2,2]>0) {nn[,2]<-c("Outcome",yb[as.numeric(nn[-1,2])]); } else {nn<-nn[,-2];}

if (!is.na(colvname)) {nn[,1]<-c(vlabel[vname==colvname],clvb_[as.numeric(nn[-1,1])]);} else {nn<-nn[,-1];}

tb<-matrix(as.numeric(tt[,c(1:4)]),ncol=4);

if (!is.na(colvname)) {tt[,1]<-c(vlabelV[vnameV==colvname],clvb[tb[-1,1]]);}

if (ny>1) {tt[,2]<-c("Outcome",yb[tb[-1,2]]);}

if (nmdl>1) {tt[,3]<-c("Model",fmlb[tb[-1,3]]);}

nrr1<-nrr-1; oo<-tt[1,]; nc<-ncol(tt)-5; nr<-nrow(tt)

for (i in 2:nr) {

if (nrr>1) {

for (j in 1:nrr1) {

if (tb[i,rord[j]]!=tb[i-1,rord[j]]) oo<-rbind(oo,c(rep(tt[i,rord[j]],5),rep(" ",nc)))

}

}

oo<-rbind(oo,tt[i,])

}

if (rord[nrr]!=4 & tt[1,rord[nrr]]!="0") {oo<-cbind(oo[,rord[nrr]],oo[,-(1:5)]);} else {oo<-oo[,-(1:4)]}

w<-c(w,"</br><table border=3>", mat2htmltable(oo), "</table>")

prnopt<-c("β (95%CI) Pvalue / OR (95%CI) Pvalue", "β (95%CI) Pvalue", "β+se / OR (95%CI) *P<0.05 **P<0.01 ***P<0.001")

# TABLE 3

## Created by EmpowerStats @ Mon, 30 Sep 24 15:34:37 +0800##

#******************** Regarding ALL Following R Code ********************

#*** COPYRIGHT (c) 2010, 2021 X&Y Solutions, ALL RIGHT RESERVED ***********

#******************* www.EmpowerStats.com *********************************

#**************************************************************************

Sys.setlocale(category = 'LC_ALL', locale = 'English_United States.1252');

.libPaths(file.path(R.home(),'library'));

library(doBy);

options(timeout=600);

library(plotrix);

library(stringi);

library(stringr);

library(survival);

library(rms);

library(nnet);

library(car);

library(mgcv);

pdfwd<-6; pdfht<-6;

load('C:/EmpowerRCH/Analysis/cmdcrptyg/202409230.Rdata');

if (length(which(ls()=='EmpowerStatsR'))==0) EmpowerStatsR<-get(ls()[1]);

names(EmpowerStatsR)<-toupper(names(EmpowerStatsR));

originalVNAME<-names(EmpowerStatsR);

ofname<-'cmdcrptyg_9_tbl';

vname<-c(NA,'GENDER','GENDER.0','GENDER.1','AGE','MARRY','MARRY.0','MARRY.1','EDU','EDU.1','EDU.2','EDU.3','EDU.4','RURAL','RURAL.0','RURAL.1','DRINK','DRINK.0','DRINK.1','DRINK.2','DRINK.3','SMOKE','SMOKE.0','SMOKE.1','SMOKE.2','SMOKE.3','LIVERE','LIVERE.0','LIVERE.1','KIDNEYE','KIDNEYE.0','KIDNEYE.1','SLEEP','CRP2','CRP2.0','CRP2.1','TYG2','TYG2.0','TYG2.1','RMS','CMD','CMD.0','CMD.1','BL_BUN','BL_CREA','BL_CHO','BL_UA','BL_HGB')[-1];

vlabel<-c(NA,'Gender',' female',' male','Age','marry',' other',' married','Education',' Primary school below',' Primary school',' middle school',' High school and above','residence',' municipalities',' countryside','DRINK',' 0',' 1',' 2',' 3','SMOKE',' 0',' 1',' 2',' 3','Liver Diseases',' no',' yes','Kidney Disease',' no',' yes','Sleeping time','CRP2',' 0',' 1','TYG2',' 0',' 1','RMS','CMD',' 0',' 1','Blood Urea Nitrogen (BUN) (mg/dl)urea nitrogen','Creatinine (mg/dl)','Total Cholesterol (mg/dl) Total Cholesterol','Uric Acid(mg/dl)','Hemoglobin (g/dl)')[-1];

varused4this <- c('GENDER','AGE','MARRY','EDU','RURAL','DRINK','SMOKE','LIVERE','KIDNEYE','SLEEP','CRP2','TYG2','RMS','CMD','BL_BUN','BL_CREA','BL_CHO','BL_UA','BL_HGB');

pkgs<-c('gdata','geepack','mgcv');

for (g in pkgs) {

if (!(g %in% rownames(installed.packages()))) install.packages(g,repos='https://cloud.r-project.org');

}

library(gdata);

library(geepack);

library(mgcv);

WD <- EmpowerStatsR; rm(EmpowerStatsR); gc();

title<-'多个回归方程';

wd.subset='';

weights.var<- NA;

yvname<-c('CMD');

ydist<-c('binomial');

ylink<-c('logit');

ylv<-c(2);

avname<-c('GENDER','AGE');

saf<-c(0,0);

alv<-c(2,0);

svname<-c('GENDER','AGE','MARRY','EDU','RURAL','DRINK','SMOKE','LIVERE','KIDNEYE','SLEEP','RMS','BL_BUN','BL_CREA','BL_CHO','BL_UA','BL_HGB');

sdf<-c(0,0,0,0,0,0,0,0,0,0,0,0,0,0,0,0);

slv<-c(2,0,2,4,2,4,4,2,2,0,0,0,0,0,0,0);

prn<-1;

xvname<-c('CRP2');

sxf<-c(0);

xlv<-c(2);

par1<-1;

chk<- 0;

cox<- 0;

timevar<- NA;

vname.start<- NA;

subjvname<- NA;

gee.TYPE<-NA;

bvar<- NA;

colvname<-'TYG2'; colvlv<- 2;

par3<-1;

dec<-1;

##R package## gdata geepack mgcv ##R package##;

pvformat<-function(p,dec) {

pp <- sprintf(paste("%.",dec,"f",sep=""),as.numeric(p))

if (is.matrix(p)) {pp<-matrix(pp, nrow=nrow(p)); colnames(pp)<-colnames(p);rownames(pp)<-rownames(p);}

lw <- paste("<",substr("0.00000000000",1,dec+1),"1",sep="");

pp[as.numeric(p)<(1/10^dec)]<-lw

return(pp)

}

numfmt<-function(p,dec) {

if (is.list(p)) p<-as.matrix(p)

pp <- sprintf(paste("%.",dec,"f",sep=""),as.numeric(p))

if (is.matrix(p)) {pp<-matrix(pp, nrow=nrow(p));colnames(pp)<-colnames(p);rownames(pp)<-rownames(p);}

pp[as.numeric(p)>10000000]<- "inf."

pp[is.na(p) | gsub(" ","",p)==""]<- ""

pp[p=="-Inf"]<-"-Inf"

pp[p=="Inf"]<-"Inf"

return(pp)

}

varstats<-function(var,vlvl,dec) {

if (length(vlvl)==1 & vlvl[1]==0) {

return(paste(numfmt(mean(var,na.rm=TRUE),dec),numfmt(sd(var,na.rm=TRUE),dec),sep="+"))

} else {

a<-table(var)

b<-matrix(paste(a, " (", numfmt(a/sum(a)*100,dec), "%)",sep=""),ncol=1)

return(c(" ",b[match(vlvl,names(a))]))

}

}

mat2htmltable<-function(mat) {

t1<- apply(mat,1,function(z) paste(z,collapse="</td><td>"))

t2<- paste("<tr><td>",t1,"</td></tr>")

return(paste(t2,collapse=" "))

}

setgam<-function(fml,yi) {

if (ydist[yi]=="") ydist[yi]<-"gaussian"

if (ydist[yi]=="exact") ydist[yi]<-"binomial"

if (ydist[yi]=="breslow") ydist[yi]<-"binomial"

if (ydist[yi]=="gaussian") mdl<-try(gam(formula(fml),weights=wdtmp$weights,data=wdtmp, family=gaussian(link="identity")))

if (ydist[yi]=="binomial") mdl<-try(gam(formula(fml),weights=wdtmp$weights,data=wdtmp, family=binomial(link="logit")))

if (ydist[yi]=="poisson") mdl<-try(gam(formula(fml),weights=wdtmp$weights,data=wdtmp, family=poisson(link="log")))

if (ydist[yi]=="gamma") mdl<-try(gam(formula(fml),weights=wdtmp$weights,data=wdtmp, family=Gamma(link="inverse")))

if (ydist[yi]=="negbin") mdl<-try(gam(formula(fml),weights=wdtmp$weights,data=wdtmp, family=negbin(c(1,10), link="log")))

return(mdl)

}

setgee<-function(fml,yi) {

if (ydist[yi]=="") ydist[yi]<-"gaussian"

if (ydist[yi]=="exact") ydist[yi]<-"binomial"

if (ydist[yi]=="breslow") ydist[yi]<-"binomial"

if (ydist[yi]=="gaussian") md<-try(geeglm(formula(fml),id=wdtmp[,subjvname],corstr=gee.TYPE,family="gaussian",weights=wdtmp$weights,data=wdtmp))

if (ydist[yi]=="binomial") md<-try(geeglm(formula(fml),id=wdtmp[,subjvname],corstr=gee.TYPE,family="binomial",weights=wdtmp$weights,data=wdtmp))

if (ydist[yi]=="poisson") md<-try(geeglm(formula(fml),id=wdtmp[,subjvname],corstr=gee.TYPE,family="poisson",weights=wdtmp$weights,data=wdtmp))

if (ydist[yi]=="gamma") md<-try(geeglm(formula(fml),id=wdtmp[,subjvname],corstr=gee.TYPE,family="Gamma",weights=wdtmp$weights,data=wdtmp))

if (ydist[yi]=="negbin") md<-try(geeglm.nb(formula(fml),id=wdtmp[,subjvname],corstr=gee.TYPE,weights=wdtmp$weights,data=wdtmp))

return(md)

}

setglm<-function(fml,yi) {

if (ydist[yi]=="") ydist[yi]<-"gaussian"

if (ydist[yi]=="exact") ydist[yi]<-"binomial"

if (ydist[yi]=="breslow") ydist[yi]<-"binomial"

if (ydist[yi]=="gaussian") md<-try(glm(formula(fml),family="gaussian",weights=wdtmp$weights,data=wdtmp))

if (ydist[yi]=="binomial") md<-try(glm(formula(fml),family="binomial",weights=wdtmp$weights,data=wdtmp))

if (ydist[yi]=="poisson") md<-try(glm(formula(fml),family="poisson",weights=wdtmp$weights,data=wdtmp))

if (ydist[yi]=="gamma") md<-try(glm(formula(fml),family="Gamma",weights=wdtmp$weights,data=wdtmp))

if (ydist[yi]=="negbin") md<-try(glm.nb(formula(fml),weights=wdtmp$weights,data=wdtmp))

return(md)

}

mdl2oo<-function(mdl, xxname, opt) {

if (is.na(mdl[[1]][1])) return(list(rep("",times=length(xxname)),""))

if (substr(mdl[[1]][1],1,5)=="Error") return(list(rep("",times=length(xxname)),""))

gs<-summary(mdl); print(mdl$formula); print(gs)

if (opt=="gam") {gsparm <- gs$p.table;tmpn<-gs$n;

} else {gsparm <- gs$coefficients;tmpn <- sum(gs$df[c(1,2)]);}

gsp<-gsparm[match(xxname,rownames(gsparm)),]

if (length(xxname)==1) {beta<-gsp[1]; se<-gsp[2]; pv<-gsp[4];

} else {beta<-gsp[,1]; se<-gsp[,2]; pv<-gsp[,4]; }

ci1<- beta-1.96*se; ci2<- beta+1.96*se

pvx<-substr(rep("****",length(pv)),1,(pv<=0.05)+(pv<=0.01)+(pv<=0.001))

if (colprn==3) {pvv<-pvx;} else {pvv<-pvformat(pv,dec+2);}

if ((colprn!=2) & (gs$family[[2]]=="log" | gs$family[[2]]=="logit")) {

o1<-paste(numfmt(exp(beta),dec)," (",numfmt(exp(ci1),dec),", ",numfmt(exp(ci2),dec),")",sep="")

} else {

if (colprn<3) {o1<-paste(numfmt(beta,dec), " (",numfmt(ci1,dec),", ",numfmt(ci2,dec),")",sep="")

} else {o1<-paste(numfmt(beta,dec), "+",numfmt(se,dec),sep="");}

}

o1<-paste(o1,pvv); o1[is.na(beta)]<-NA

if (length(xxname)>1) {

if (gs$family[[2]]=="log" | gs$family[[2]]=="logit") {

o1[is.na(o1) & substr(xxname,1,7)=="factor("]<-"1.0"

} else {o1[is.na(o1) & substr(xxname,1,7)=="factor("]<-"0";}

o1[is.na(o1)]<-"";

}

return(list(o1,tmpn))

}

recodevar <- function (var,oldcode,newcode) {

tmp.v <- var

nc.tmp <- length(oldcode)

for (i in (1:nc.tmp)) {tmp.v[(var==oldcode[i])]=newcode[i]}

if (is.factor(tmp.v)) {tmp.v1<-as.numeric(as.character(tmp.v))} else {tmp.v1<-as.numeric(tmp.v)}

rm(tmp.v); return(tmp.v1)

}

rankvar <- function(var, num) {

qprobs <- 1/num

if (num>2) {for (i in (2:(num-1))) {qprobs <- c(qprobs, 1/num * i) } }

outvar <- rep(0, times=length(var))

outvar[is.na(var)] <- NA

cutpoints <- quantile(var,probs=qprobs, na.rm=TRUE)

for (k in (1:length(cutpoints))) { outvar[var>=cutpoints[k]] <- k; }

return(outvar)

}

removeNA<-function(i,j,m,wdf) {

vvv<-c(yvname[i],adjvv[[m]],subjvname,colvname,bvar,vname.start,timevar);

if (j<=nx) {vvv<-c(vvv,xvname[j]);} else {vvv<-c(vvv,xvname);}

vvv<-vvv[!is.na(vvv)]; vvv<-vvv[vvv>" "]

tmp<-is.na(wdf[,vvv]);

return(wdf[apply(tmp,1,sum)==0,])

}

if (!is.na(weights.var)) {weights<-WD[,weights.var];} else {weights<-1;}

WD<-cbind(WD,weights);

vlabelN<-(substr(vlabel,1,1)==" ");

vlabelZ<-vlabel[vlabelN];vlabelV<-vlabel[!vlabelN]

vnameV<-vname[!vlabelN];vnameZ<-vname[vlabelN]

w<-c("<!DOCTYPE html><html lang='zh'><head><meta charset='utf-8'></head><body>")

w<-c(w,paste("<h2>", title, "</h2>"))

if (length(avname)>0) {

if (sum((saf=="s" | saf=="S") & alv>0)>0) w<-c(w,"</br>Spline smoothing only applies for continuous variables")

if (!is.na(subjvname) & (sum((saf=="s" | saf=="S") & alv==0)>0)) w<-c(w,"</br>Generalized estimate equation could not be used with spline smoothing terms")

}

if (length(svname)>0) {

if (sum((sdf=="s" | sdf=="S") & slv>0)>0) w<-c(w,"</br>Spline smoothing only applies for continuous variables")

if (!is.na(subjvname) & (sum((sdf=="s" | sdf=="S") & slv==0)>0)) w<-c(w,"</br>Generalized estimate equation could not be used with spline smoothing terms")

}

allvname<-c(yvname,xvname,colvname,bvar,avname,svname,subjvname,vname.start,timevar,"weights");

allvname<-allvname[!is.na(allvname)]

WD<-WD[,allvname];

if (!is.na(subjvname)) WD<-WD[order(WD[,subjvname]),]

sxf<-as.numeric(sxf);sxf[is.na(sxf)]<-0;

if (sum(sxf>1 & xlv>0)>0) w<-c(w,"Categorizing only applies to continuous variables");

if (sum(sxf>1 & xlv==0)>0) {

t.xname<-NA;t.xlv<-NA; nx<-length(xvname)

for (i in 1:nx) {

if (sxf[i]>1 & xlv[i]==0) {

tmp.Xi<- rankvar(WD[,xvname[i]],sxf[i])

tmp.newcode <- tapply(WD[,xvname[i]],tmp.Xi,function(z) median(z,na.rm=TRUE))

tmp.low <- tapply(WD[,xvname[i]],tmp.Xi,function(z) min(z,na.rm=TRUE))

tmp.upp <- tapply(WD[,xvname[i]],tmp.Xi,function(z) max(z,na.rm=TRUE))

tmp.Xi2<- recodevar(tmp.Xi,(1:sxf[i])-1,tmp.newcode)

tmp.Xi<-cbind(tmp.Xi,tmp.Xi2)

tmp.NM<-paste(xvname[i],c("grp","grp.cont"),sep=".")

colnames(tmp.Xi)<-tmp.NM

WD<-cbind(WD,tmp.Xi)

t.xname<-c(t.xname,tmp.NM)

t.xlv<-c(t.xlv,sxf[i],0)

vnameV<-c(vnameV,tmp.NM)

vlabelV<-c(vlabelV,paste(vlabelV[vnameV==xvname[i]],c("group","group trend")))

vnameZ<-c(vnameZ,paste(tmp.NM[1],(1:sxf[i])-1,sep="."))

vlabelZ<-c(vlabelZ,paste(tmp.low,"-",tmp.upp))

} else {

t.xname<-c(t.xname,xvname[i]); t.xlv<-c(t.xlv,xlv[i])

}

}

xvname<-t.xname[-1]; xlv<-t.xlv[-1];

}

if (!is.na(subjvname)) {

if (length(avname)>0) saf<-rep(0,length(saf));

if (length(svname)>0) sdf<-rep(0,length(sdf));

WD<-WD[order(WD[,subjvname]),];

}

ny=length(yvname); nx=length(xvname);

xb<-vlabelV[match(xvname,vnameV)]; xb[is.na(xb)]<-xvname[is.na(xb)]

yb<-vlabelV[match(yvname,vnameV)]; yb[is.na(yb)]<-yvname[is.na(yb)]

xvname_ <- xvname

xvname_[xlv>0]<-paste("factor(",xvname[xlv>0],")",sep="")

xxname_<-list(NA); xxlbl_<-list(NA); xxlvl_<-list(NA)

for (j in (1:nx)) {

if (xlv[j]==0) {

xxname_[[j+1]]<-xvname[j];xxlbl_[[j+1]]<-xb[j];xxlvl_[[j+1]]<-0

} else {

xxlvl_[[j+1]]<-levels(factor(WD[,xvname[j]]))

tmp<-paste(xvname[j],".",xxlvl_[[j+1]],sep="")

xxlbl_[[j+1]]<-c(xb[j],vlabelZ[match(tmp,vnameZ)])

xxlbl_[[j+1]]<-paste(c("",rep("  ",length(xxlbl_[[j+1]])-1)),xxlbl_[[j+1]])

xxname_[[j+1]]<-c(xvname[j],paste("factor(",xvname[j],")",xxlvl_[[j+1]],sep=""))

}

}

xxname_<-xxname_[-1]; xxlbl_<-xxlbl_[-1]; xxlvl_<-xxlvl_[-1];

if (nx==1) par1<-1;

if (nx!=length(svname) & par1==4) par1<-1;

if (is.na(par1) | par1<1) par1<-1;

if (par1>1 & par1 < 4) {

tmp1<-xxname_[[1]]; tmp2<-xxlbl_[[1]]

for (j in 2:nx) {tmp1<-c(tmp1,xxname_[[j]]); tmp2<-c(tmp2,xxlbl_[[j]]);}

xxname_[[nx+1]]<-tmp1; xxlbl_[[nx+1]]<-tmp2;

xvname_<-c(xvname_,paste(xvname_,collapse="+"))

}

contx<-(sum(xlv>0)==0)

if (par1==3 & !is.na(bvar)) {w<-c(w,"</br>Column stratified variable was ignored"); bvar<-NA; bvname<-NA;}

fmlm<-" "; fmlb<-"Non-adjusted"; tmp<-""; adjvv<-list(NA); adjvb<-"None";

fmlm4<-list(rep("",nx))

fmlp<-ifelse(!is.na(subjvname), "gee", "glm");

na=0; avb=""; smoothav<-0; nadjm<-0

if (length(avname)>0) {

na<-length(avname)

avb<-vlabelV[match(avname,vnameV)];

avname_ <- avname

smoothavi<-((saf=="s" | saf=="S") & alv==0)

smoothav<-sum(smoothavi)

smoothavname<-avname[smoothavi]

avname_[smoothavi]<-paste("s(",avname[smoothavi],")",sep="")

avb1<-avb

avb1[smoothavi]<-paste(avb[smoothavi],"(Smooth)",sep="")

avname_[alv>0]<-paste("factor(",avname[alv>0],")",sep="")

fmlm<-c(fmlm,paste("+",paste(avname_,collapse="+")))

fmlb<-c(fmlb,"Adjust")

nadjm<-nadjm+1; tmp<-c(tmp,"I"); adjvv[[nadjm+1]]<-avname;

adjvb<-c(adjvb, paste(avb1, collapse="; "))

fmlp<-c(fmlp,ifelse(!is.na(subjvname), "gee", ifelse(smoothav>0, "gam", "glm")))

fmlm4<-c(fmlm4, list(rep("",nx)))

}

ns=0; svb=""; smoothsv<-0

if (length(svname)>0) {

svb<-vlabelV[match(svname,vnameV)];

svname_ <- svname

smoothsvi<-((sdf=="s" | sdf=="S") & slv==0)

smoothsv<-sum(smoothsvi)

smoothsvname<-svname[smoothsvi]

svname_[smoothsvi]<-paste("s(",svname[smoothsvi],")",sep="")

svb1<-svb

svb1[smoothsvi]<-paste(svb[smoothsvi],"(Smooth)",sep="")

svname_[slv>0]<-paste("factor(",svname[slv>0],")",sep="")

if (par1==4) {

fmlm<-c(fmlm, paste(fmlm[length(fmlm)],"+"));

fmlm4<-c(fmlm4, list(svname_));

} else {

fmlm<-c(fmlm,paste("+",paste(svname_,collapse="+")));

fmlm4<-c(fmlm4, list(rep("",nx)));

}

fmlb<-c(fmlb,"Adjust")

nadjm<-nadjm+1; tmp<-c(tmp,"II"); adjvv[[nadjm+1]]<-svname

adjvb2 <- paste(svb1, collapse="; ")

if (par1 == 4) {

if (avb>"") {adjvb2<-paste(avb1, "AND one in (", adjvb2,") respectively for each exposure X");

} else { adjvb2<-paste("ONE of (", adjvb2,") respectively for each exposure X");}

}

adjvb<-c(adjvb, adjvb2)

fmlp<-c(fmlp,ifelse(!is.na(subjvname), "gee", ifelse(smoothsv>0, "gam", "glm")))

}

if (chk==0 & length(fmlm)>1) {

fmlm<-fmlm[-1]; fmlb<-fmlb[-1]; tmp<-tmp[-1]; adjvv<-adjvv[-1]; adjvb<-adjvb[-1]; fmlp<-fmlp[-1]; fmlm4<-fmlm4[-1];

}

if (nadjm>1) fmlb<-paste(fmlb,tmp)

nmdl<-length(fmlm)

if (is.na(bvar) & !is.na(colvname) & nmdl==1 & par1!=3) {if ((ny==1) | (nx==1 & contx)) {bvar<-colvname; colvname<-NA;}}

if (is.na(colvname)) {

nclv<-1; clvb<-"Total"; clvb_<-"Total"

} else {

clv<-levels(factor(WD[,colvname])); nclv<-length(clv)+1

clvb_<-vlabelZ[match(paste(colvname,".",clv,sep=""),vnameZ)]; clvb_[is.na(clvb_)]<-clv[is.na(clvb_)];

clvb<-c(paste(vlabelV[vnameV==colvname],clvb_,sep="="),"Total");

clvb_<-c(clvb_,"Total")

WD<-WD[!is.na(WD[,colvname]),]

}

if (is.na(bvar)) {

blvb<-"Total"; blvb_<-"Total"

} else {

blv<-levels(factor(WD[,bvar])); nblv<-length(blv)+1

blvb_<-vlabelZ[match(paste(bvar,".",blv,sep=""),vnameZ)]; blvb_[is.na(blvb_)]<-blv[is.na(blvb_)];

blvb<-c(paste(vlabelV[vnameV==bvar],blvb_,sep="="),"Total");

blvb_<-c(blvb_,"Total")

WD<-WD[!is.na(WD[,bvar]),]

}

aa<-c(1,2,3,4)

for (i in 1:4) {

for (j in c(1:4)[-i]) {

for (k in c(1:4)[-c(i,j)]) aa<-rbind(aa,c(i,j,k,c(1:4)[-c(i,j,k)]))

}

}

colprn<-prn;

if (is.na(par3) | par3<1) par3<-1

rord<-aa[par3,]

if (!is.na(bvar)) {prn<-"S";

} else {

if (par3>1) {

rordc<-ifelse(rord[4]==1,3,4)

prn<-c("G","Y","M","X")[rord[rordc]]

if (prn=="X") {

if (!contx & nx>1) prn<-ifelse(nmdl>1, "M", ifelse(ny>nx & contx, "X", "Y"))

if (!contx & nx==1) prn<-"CX"

if (par1==2) {tmp<-ifelse(rordc==4,ifelse(rord[3]==1,2,3),2); prn<-c("G","Y","M","X")[rord[tmp]];}

}

} else {

prn<-ifelse(nmdl>1, "M", ifelse(ny>nx & contx, "X", "Y"))

if (par1==2) prn<-ifelse(nmdl>1, "M", "Y")

}

if (par1==3) prn<-"UM"

}

sink(paste(ofname,".lst",sep=""))

if (par1==2) {xbgn<-nx+1; xend<-nx+1;} else {xbgn<-1; xend<-nx;}

if (prn=="Y") {

tt<-c(0,0,0,0,"Exposure",yb); nn<-c(0,0,0,0,yb);

for (k in (1:nclv)) {

wdtmp0<-WD;

if (!is.na(colvname)) {

if (k<nclv) wdtmp0<-WD[WD[,colvname]==clv[k],];

print(paste("Stratified by",colvname, ":", clvb[k]))

}

for (m in 1:nmdl) {

for (j in (xbgn:xend)) {

colj<-cbind(k,0,m,j,xxlbl_[[j]])

nnj <-c(k,0,m,j)

for (i in (1:ny)) {

if (par1==4) {fml<-paste(yvname[i],"~",xvname_[j],fmlm[m],fmlm4[[m]][j]);} else {fml<-paste(yvname[i],"~",xvname_[j],fmlm[m]);}

wdtmp<-removeNA(i,j,m,wdtmp0)

if (!is.na(colvname)) {if (k==nclv) fml<-paste(fml,"+factor(",colvname,")",sep="");}

if (fmlp[m]=="gam") tmp.mdl<-setgam(fml,i)

if (fmlp[m]=="gee") tmp.mdl<-setgee(fml,i)

if (fmlp[m]=="glm") tmp.mdl<-setglm(fml,i)

tmpooi<-mdl2oo(tmp.mdl,xxname_[[j]],fmlp[m])

colj<-cbind(colj,tmpooi[[1]]); nnj<-c(nnj,tmpooi[[2]])

}

tt<-rbind(tt,colj); nn<-rbind(nn,nnj)

}

}

}

}

if (prn=="S") {

tt<-c(0,0,0,0,"Exposure",blvb); nn<-c(0,0,0,0,blvb);

for (k in (1:nclv)) {

wdtmp0<-WD;

if (!is.na(colvname)) {

if (k<nclv) wdtmp0<-WD[WD[,colvname]==clv[k],];

print(paste("Stratified by",colvname, ":", clvb[k]))

}

for (i in (1:ny)) {

for (m in 1:nmdl) {

for (j in (xbgn:xend)) {

colj<-cbind(k,i,m,j,xxlbl_[[j]]);

nnj <- c(k,i,m,j)

for (b in (1:nblv)) {

print(paste("Stratified by",bvar, ":", blvb[b]))

if (par1==4) {fml<-paste(yvname[i],"~",xvname_[j],fmlm[m],fmlm4[[m]][j]);} else {fml<-paste(yvname[i],"~",xvname_[j],fmlm[m]);}

if (b<nblv) {wdtmp1<-wdtmp0[wdtmp0[,bvar]==blv[b],];} else {wdtmp1<-wdtmp0; fml<-paste(fml,"+factor(",bvar,")",sep="");}

wdtmp<-removeNA(i,j,m,wdtmp1)

if (!is.na(colvname)) {if (k==nclv) fml<-paste(fml,"+factor(",colvname,")",sep="");}

if (fmlp[m]=="gam") tmp.mdl<-setgam(fml,i)

if (fmlp[m]=="gee") tmp.mdl<-setgee(fml,i)

if (fmlp[m]=="glm") tmp.mdl<-setglm(fml,i)

tmpooi<-mdl2oo(tmp.mdl,xxname_[[j]],fmlp[m])

colj<-cbind(colj,tmpooi[[1]]); nnj<-c(nnj,tmpooi[[2]])

}

tt<-rbind(tt,colj); nn<-rbind(nn,nnj)

}

}

}

}

}

if (prn=="M") {

tt<-c(0,0,0,0,"Exposure",fmlb); nn<-c(0,0,0,0,fmlb)

for (k in (1:nclv)) {

wdtmp0<-WD;

if (!is.na(colvname)) {

if (k<nclv) wdtmp0<-WD[WD[,colvname]==clv[k],];

print(paste("Stratified by",colvname, ":", clvb[k]))

}

for (i in 1:ny) {

for (j in xbgn:xend) {

colj<-cbind(k,i,0,j,xxlbl_[[j]]); nnj<-c(k,i,0,j)

for (m in 1:nmdl) {

if (par1==4) {fml<-paste(yvname[i],"~",xvname_[j],fmlm[m],fmlm4[[m]][j]);} else {fml<-paste(yvname[i],"~",xvname_[j],fmlm[m]);}

wdtmp<-removeNA(i,j,m,wdtmp0)

if (!is.na(colvname)) {if (k==nclv) fml<-paste(fml,"+factor(",colvname,")",sep="");}

if (fmlp[m]=="gam") tmp.mdl<-setgam(fml,i)

if (fmlp[m]=="gee") tmp.mdl<-setgee(fml,i)

if (fmlp[m]=="glm") tmp.mdl<-setglm(fml,i)

tmpooi<-mdl2oo(tmp.mdl,xxname_[[j]],fmlp[m])

colj<-cbind(colj,tmpooi[[1]]); nnj<-c(nnj,tmpooi[[2]])

}

tt<-rbind(tt,colj); nn<-rbind(nn,nnj)

}

}

}

}

if (prn=="X") {

tt<-c(0,0,0,0,"Outcome",xb); nn<-c(0,0,0,0,xb);

for (k in (1:nclv)) {

wdtmp0<-WD;

if (!is.na(colvname)) {

if (k<nclv) wdtmp0<-WD[WD[,colvname]==clv[k],];

print(paste("Stratified by",colvname, ":", clvb[k]))

}

for (m in 1:nmdl) {

for (i in (1:ny)) {

colj<-cbind(k,i,m,0,yb[i])

nnj <-c(k,i,m,0)

for (j in (1:nx)) {

if (par1==4) {fml<-paste(yvname[i],"~",xvname_[j],fmlm[m],fmlm4[[m]][j]);} else {fml<-paste(yvname[i],"~",xvname_[j],fmlm[m]);}

wdtmp<-removeNA(i,j,m,wdtmp0)

if (!is.na(colvname)) {if (k==nclv) fml<-paste(fml,"+factor(",colvname,")",sep="");}

if (fmlp[m]=="gam") tmp.mdl<-setgam(fml,i)

if (fmlp[m]=="gee") tmp.mdl<-setgee(fml,i)

if (fmlp[m]=="glm") tmp.mdl<-setglm(fml,i)

tmpooi<-mdl2oo(tmp.mdl,xxname_[[j]],fmlp[m])

colj<-cbind(colj,tmpooi[[1]]); nnj<-c(nnj,tmpooi[[2]])

}

tt<-rbind(tt,colj); nn<-rbind(nn,nnj)

}

}

}

}

if (prn=="CX") {

tt<-c(0,0,0,0,"Outcome",xxlbl_[[1]][-1]); nn<-c(0,0,0,0,xb[1]);

nxl<-length(xxlbl_[[1]])-1

for (k in (1:nclv)) {

wdtmp0<-WD;

if (!is.na(colvname)) {

if (k<nclv) wdtmp0<-WD[WD[,colvname]==clv[k],];

print(paste("Stratified by",colvname, ":", clvb[k]))

}

for (m in 1:nmdl) {

for (i in (1:ny)) {

colj<-c(k,i,m,0,yb[i])

nnj <-c(k,i,m,0)

fml<-paste(yvname[i],"~",xvname_[1],fmlm[m]);

wdtmp<-removeNA(i,1,m,wdtmp0)

if (!is.na(colvname)) {if (k==nclv) fml<-paste(fml,"+factor(",colvname,")",sep="");}

if (fmlp[m]=="gam") tmp.mdl<-setgam(fml,i)

if (fmlp[m]=="gee") tmp.mdl<-setgee(fml,i)

if (fmlp[m]=="glm") tmp.mdl<-setglm(fml,i)

tmpooi<-mdl2oo(tmp.mdl,xxname_[[1]],fmlp[m])

colj<-c(colj,tmpooi[[1]][-1]); nnj<-c(nnj,tmpooi[[2]])

tt<-rbind(tt,colj); nn<-rbind(nn,nnj)

}

}

}

}

if (prn=="UM") {

tt<-c(0,0,0,0,"Exposure","Univariable","Multivariable"); nn<-c(0,0,0,0,"Univariable","Multivariable")

for (k in (1:nclv)) {

wdtmp0<-WD;

if (!is.na(colvname)) {

if (k<nclv) wdtmp0<-WD[WD[,colvname]==clv[k],];

print(paste("Stratified by",colvname, ":", clvb[k]))

}

for (i in 1:ny) {

for (m in 1:nmdl) {

colm<-rep(NA,6); nnm<-rep(NA,5)

for (j in 1:(nx+1)) {

colj<-cbind(k,i,m,j,xxlbl_[[j]]); nnj<-c(k,i,m,j)

if (par1==4) {fml<-paste(yvname[i],"~",xvname_[j],fmlm[m],fmlm4[[m]][j]);} else {fml<-paste(yvname[i],"~",xvname_[j],fmlm[m]);}

wdtmp<-removeNA(i,j,m,wdtmp0)

if (!is.na(colvname)) {if (k==nclv) fml<-paste(fml,"+factor(",colvname,")",sep="");}

if (fmlp[m]=="gam") tmp.mdl<-setgam(fml,i)

if (fmlp[m]=="gee") tmp.mdl<-setgee(fml,i)

if (fmlp[m]=="glm") tmp.mdl<-setglm(fml,i)

tmpooi<-mdl2oo(tmp.mdl,xxname_[[j]],fmlp[m])

colj<-cbind(colj,tmpooi[[1]]); nnj<-c(nnj,tmpooi[[2]])

if (j<=nx) {colm<-rbind(colm,colj); nnm<-rbind(nnm,nnj);

} else {colm<-cbind(colm[-1,],tmpooi[[1]]); nnm<-cbind(nnm[-1,],tmpooi[[2]]);}

}

tt<-rbind(tt,colm); nn<-rbind(nn,nnm)

}

}

}

}

sink()

if (!contx & prn!="CX") rord<-c(rord[rord!=4],4)

if (prn=="X" | prn=="CX") rord<-rord[rord!=4]

if (prn=="Y") rord<-rord[rord!=2]

if (prn=="M") rord<-rord[rord!=3]

if (nx==1 & contx) rord<-rord[rord!=4]

if (nmdl==1) rord<-rord[rord!=3]

if (ny==1) rord<-rord[rord!=2]

if (is.na(colvname)) rord<-rord[rord!=1]

if (length(rord)==0) rord<-1

nrr<-length(rord)

for (i in nrr:1) {nn<-nn[order(as.numeric(nn[,rord[i]])),];tt<-tt[order(as.numeric(tt[,rord[i]])),];}

if (nn[2,4]>0) {nn[,4]<-c("Exposure",xb[as.numeric(nn[-1,4])]); } else {nn<-nn[,-4];}

if (nn[2,3]>0) {nn[,3]<-c("Model",fmlb[as.numeric(nn[-1,3])]); } else {nn<-nn[,-3];}

if (nn[2,2]>0) {nn[,2]<-c("Outcome",yb[as.numeric(nn[-1,2])]); } else {nn<-nn[,-2];}

if (!is.na(colvname)) {nn[,1]<-c(vlabel[vname==colvname],clvb_[as.numeric(nn[-1,1])]);} else {nn<-nn[,-1];}

tb<-matrix(as.numeric(tt[,c(1:4)]),ncol=4);

if (!is.na(colvname)) {tt[,1]<-c(vlabelV[vnameV==colvname],clvb[tb[-1,1]]);}

if (ny>1) {tt[,2]<-c("Outcome",yb[tb[-1,2]]);}

if (nmdl>1) {tt[,3]<-c("Model",fmlb[tb[-1,3]]);}

nrr1<-nrr-1; oo<-tt[1,]; nc<-ncol(tt)-5; nr<-nrow(tt)

for (i in 2:nr) {

if (nrr>1) {

for (j in 1:nrr1) {

if (tb[i,rord[j]]!=tb[i-1,rord[j]]) oo<-rbind(oo,c(rep(tt[i,rord[j]],5),rep(" ",nc)))

}

}

oo<-rbind(oo,tt[i,])

}

if (rord[nrr]!=4 & tt[1,rord[nrr]]!="0") {oo<-cbind(oo[,rord[nrr]],oo[,-(1:5)]);} else {oo<-oo[,-(1:4)]}

w<-c(w,"</br><table border=3>", mat2htmltable(oo), "</table>")

prnopt<-c("β (95%CI) Pvalue / OR (95%CI) Pvalue", "β (95%CI) Pvalue", "β+se / OR (95%CI) *P<0.05 **P<0.01 ***P<0.001")

# FIGURE 3

## Created by EmpowerStats @ Mon, 30 Sep 24 15:50:49 +0800##

#******************** Regarding ALL Following R Code ********************

#*** COPYRIGHT (c) 2010, 2021 X&Y Solutions, ALL RIGHT RESERVED ***********

#******************* www.EmpowerStats.com *********************************

#**************************************************************************

Sys.setlocale(category = 'LC_ALL', locale = 'English_United States.1252');

.libPaths(file.path(R.home(),'library'));

library(doBy);

options(timeout=600);

library(plotrix);

library(stringi);

library(stringr);

library(survival);

library(rms);

library(nnet);

library(car);

library(mgcv);

pdfwd<-6; pdfht<-6;

load('C:/EmpowerRCH/Analysis/cmdcrptyg/202409230.Rdata');

if (length(which(ls()=='EmpowerStatsR'))==0) EmpowerStatsR<-get(ls()[1]);

names(EmpowerStatsR)<-toupper(names(EmpowerStatsR));

originalVNAME<-names(EmpowerStatsR);

ofname<-'cmdcrptyg_22_tbl';

attach(EmpowerStatsR);

sink(paste(ofname,'_datastep.lst',sep=''));

print('Creating new variable: AGE.C');

AGE.C<- 0+(AGE>=60)+(AGE>=70);

AGE.C[is.na(AGE)]<- NA;

summary(AGE.C);

EmpowerStatsR<-cbind(EmpowerStatsR,AGE.C);

rm(AGE.C);

sink();

vname<-c(NA,'GENDER','GENDER.0','GENDER.1','AGE','MARRY','MARRY.0','MARRY.1','EDU','EDU.1','EDU.2','EDU.3','EDU.4','RURAL','RURAL.0','RURAL.1','DRINK','DRINK.0','DRINK.1','DRINK.2','DRINK.3','SMOKE','SMOKE.0','SMOKE.1','SMOKE.2','SMOKE.3','LIVERE','LIVERE.0','LIVERE.1','KIDNEYE','KIDNEYE.0','KIDNEYE.1','SLEEP','CRP.TYG','CRP.TYG.1','CRP.TYG.2','CRP.TYG.3','CRP.TYG.4','RMS','CMD','CMD.0','CMD.1','BL_BUN','BL_CREA','BL_CHO','BL_UA','BL_HGB','AGE.C','AGE.C.0','AGE.C.1','AGE.C.2')[-1];

vlabel<-c(NA,'Gender',' female',' male','Age','marry',' other',' married','Education',' Primary school below',' Primary school',' middle school',' High school and above','residence',' municipalities',' countryside','DRINK',' 0',' 1',' 2',' 3','SMOKE',' 0',' 1',' 2',' 3','Liver Diseases',' no',' yes','Kidney Disease',' no',' yes','Sleeping time','CRP.TYG',' 1',' 2',' 3',' 4','RMS','CMD',' 0',' 1','Blood Urea Nitrogen (BUN) (mg/dl)urea nitrogen','Creatinine (mg/dl)','Total Cholesterol (mg/dl) Total Cholesterol','Uric Acid(mg/dl)','Hemoglobin (g/dl)','Age categorical',' <60',' >=60, <70',' >=70')[-1];

varused4this <- c('GENDER','AGE','MARRY','EDU','RURAL','DRINK','SMOKE','LIVERE','KIDNEYE','SLEEP','CRP.TYG','RMS','CMD','BL_BUN','BL_CREA','BL_CHO','BL_UA','BL_HGB','AGE.C');

pkgs<-c('gdata','geepack','mgcv');

for (g in pkgs) {

if (!(g %in% rownames(installed.packages()))) install.packages(g,repos='https://cloud.r-project.org');

}

library(gdata);

library(geepack);

library(mgcv);

WD <- EmpowerStatsR; rm(EmpowerStatsR); gc();

title<-'多个回归方程';

wd.subset='';

weights.var<- NA;

yvname<-c('CMD');

ydist<-c('binomial');

ylink<-c('logit');

ylv<-c(2);

avname<-c('GENDER','AGE');

saf<-c(0,0);

alv<-c(2,0);

svname<-c('GENDER','AGE','MARRY','EDU','RURAL','DRINK','SMOKE','LIVERE','KIDNEYE','SLEEP','RMS','BL_BUN','BL_CREA','BL_CHO','BL_UA','BL_HGB');

sdf<-c(0,0,0,0,0,0,0,0,0,0,0,0,0,0,0,0);

slv<-c(2,0,2,4,2,4,4,2,2,0,0,0,0,0,0,0);

prn<-1;

xvname<-c('CRP.TYG');

sxf<-c(0);

xlv<-c(4);

par1<-1;

chk<- 0;

cox<- 0;

timevar<- NA;

vname.start<- NA;

subjvname<- NA;

gee.TYPE<-NA;

bvar<- NA;

colvname<-'AGE.C'; colvlv<- 3;

par3<-1;

dec<-1;

##R package## gdata geepack mgcv ##R package##;

pvformat<-function(p,dec) {

pp <- sprintf(paste("%.",dec,"f",sep=""),as.numeric(p))

if (is.matrix(p)) {pp<-matrix(pp, nrow=nrow(p)); colnames(pp)<-colnames(p);rownames(pp)<-rownames(p);}

lw <- paste("<",substr("0.00000000000",1,dec+1),"1",sep="");

pp[as.numeric(p)<(1/10^dec)]<-lw

return(pp)

}

numfmt<-function(p,dec) {

if (is.list(p)) p<-as.matrix(p)

pp <- sprintf(paste("%.",dec,"f",sep=""),as.numeric(p))

if (is.matrix(p)) {pp<-matrix(pp, nrow=nrow(p));colnames(pp)<-colnames(p);rownames(pp)<-rownames(p);}

pp[as.numeric(p)>10000000]<- "inf."

pp[is.na(p) | gsub(" ","",p)==""]<- ""

pp[p=="-Inf"]<-"-Inf"

pp[p=="Inf"]<-"Inf"

return(pp)

}

varstats<-function(var,vlvl,dec) {

if (length(vlvl)==1 & vlvl[1]==0) {

return(paste(numfmt(mean(var,na.rm=TRUE),dec),numfmt(sd(var,na.rm=TRUE),dec),sep="+"))

} else {

a<-table(var)

b<-matrix(paste(a, " (", numfmt(a/sum(a)*100,dec), "%)",sep=""),ncol=1)

return(c(" ",b[match(vlvl,names(a))]))

}

}

mat2htmltable<-function(mat) {

t1<- apply(mat,1,function(z) paste(z,collapse="</td><td>"))

t2<- paste("<tr><td>",t1,"</td></tr>")

return(paste(t2,collapse=" "))

}

setgam<-function(fml,yi) {

if (ydist[yi]=="") ydist[yi]<-"gaussian"

if (ydist[yi]=="exact") ydist[yi]<-"binomial"

if (ydist[yi]=="breslow") ydist[yi]<-"binomial"

if (ydist[yi]=="gaussian") mdl<-try(gam(formula(fml),weights=wdtmp$weights,data=wdtmp, family=gaussian(link="identity")))

if (ydist[yi]=="binomial") mdl<-try(gam(formula(fml),weights=wdtmp$weights,data=wdtmp, family=binomial(link="logit")))

if (ydist[yi]=="poisson") mdl<-try(gam(formula(fml),weights=wdtmp$weights,data=wdtmp, family=poisson(link="log")))

if (ydist[yi]=="gamma") mdl<-try(gam(formula(fml),weights=wdtmp$weights,data=wdtmp, family=Gamma(link="inverse")))

if (ydist[yi]=="negbin") mdl<-try(gam(formula(fml),weights=wdtmp$weights,data=wdtmp, family=negbin(c(1,10), link="log")))

return(mdl)

}

setgee<-function(fml,yi) {

if (ydist[yi]=="") ydist[yi]<-"gaussian"

if (ydist[yi]=="exact") ydist[yi]<-"binomial"

if (ydist[yi]=="breslow") ydist[yi]<-"binomial"

if (ydist[yi]=="gaussian") md<-try(geeglm(formula(fml),id=wdtmp[,subjvname],corstr=gee.TYPE,family="gaussian",weights=wdtmp$weights,data=wdtmp))

if (ydist[yi]=="binomial") md<-try(geeglm(formula(fml),id=wdtmp[,subjvname],corstr=gee.TYPE,family="binomial",weights=wdtmp$weights,data=wdtmp))

if (ydist[yi]=="poisson") md<-try(geeglm(formula(fml),id=wdtmp[,subjvname],corstr=gee.TYPE,family="poisson",weights=wdtmp$weights,data=wdtmp))

if (ydist[yi]=="gamma") md<-try(geeglm(formula(fml),id=wdtmp[,subjvname],corstr=gee.TYPE,family="Gamma",weights=wdtmp$weights,data=wdtmp))

if (ydist[yi]=="negbin") md<-try(geeglm.nb(formula(fml),id=wdtmp[,subjvname],corstr=gee.TYPE,weights=wdtmp$weights,data=wdtmp))

return(md)

}

setglm<-function(fml,yi) {

if (ydist[yi]=="") ydist[yi]<-"gaussian"

if (ydist[yi]=="exact") ydist[yi]<-"binomial"

if (ydist[yi]=="breslow") ydist[yi]<-"binomial"

if (ydist[yi]=="gaussian") md<-try(glm(formula(fml),family="gaussian",weights=wdtmp$weights,data=wdtmp))

if (ydist[yi]=="binomial") md<-try(glm(formula(fml),family="binomial",weights=wdtmp$weights,data=wdtmp))

if (ydist[yi]=="poisson") md<-try(glm(formula(fml),family="poisson",weights=wdtmp$weights,data=wdtmp))

if (ydist[yi]=="gamma") md<-try(glm(formula(fml),family="Gamma",weights=wdtmp$weights,data=wdtmp))

if (ydist[yi]=="negbin") md<-try(glm.nb(formula(fml),weights=wdtmp$weights,data=wdtmp))

return(md)

}

mdl2oo<-function(mdl, xxname, opt) {

if (is.na(mdl[[1]][1])) return(list(rep("",times=length(xxname)),""))

if (substr(mdl[[1]][1],1,5)=="Error") return(list(rep("",times=length(xxname)),""))

gs<-summary(mdl); print(mdl$formula); print(gs)

if (opt=="gam") {gsparm <- gs$p.table;tmpn<-gs$n;

} else {gsparm <- gs$coefficients;tmpn <- sum(gs$df[c(1,2)]);}

gsp<-gsparm[match(xxname,rownames(gsparm)),]

if (length(xxname)==1) {beta<-gsp[1]; se<-gsp[2]; pv<-gsp[4];

} else {beta<-gsp[,1]; se<-gsp[,2]; pv<-gsp[,4]; }

ci1<- beta-1.96*se; ci2<- beta+1.96*se

pvx<-substr(rep("****",length(pv)),1,(pv<=0.05)+(pv<=0.01)+(pv<=0.001))

if (colprn==3) {pvv<-pvx;} else {pvv<-pvformat(pv,dec+2);}

if ((colprn!=2) & (gs$family[[2]]=="log" | gs$family[[2]]=="logit")) {

o1<-paste(numfmt(exp(beta),dec)," (",numfmt(exp(ci1),dec),", ",numfmt(exp(ci2),dec),")",sep="")

} else {

if (colprn<3) {o1<-paste(numfmt(beta,dec), " (",numfmt(ci1,dec),", ",numfmt(ci2,dec),")",sep="")

} else {o1<-paste(numfmt(beta,dec), "+",numfmt(se,dec),sep="");}

}

o1<-paste(o1,pvv); o1[is.na(beta)]<-NA

if (length(xxname)>1) {

if (gs$family[[2]]=="log" | gs$family[[2]]=="logit") {

o1[is.na(o1) & substr(xxname,1,7)=="factor("]<-"1.0"

} else {o1[is.na(o1) & substr(xxname,1,7)=="factor("]<-"0";}

o1[is.na(o1)]<-"";

}

return(list(o1,tmpn))

}

recodevar <- function (var,oldcode,newcode) {

tmp.v <- var

nc.tmp <- length(oldcode)

for (i in (1:nc.tmp)) {tmp.v[(var==oldcode[i])]=newcode[i]}

if (is.factor(tmp.v)) {tmp.v1<-as.numeric(as.character(tmp.v))} else {tmp.v1<-as.numeric(tmp.v)}

rm(tmp.v); return(tmp.v1)

}

rankvar <- function(var, num) {

qprobs <- 1/num

if (num>2) {for (i in (2:(num-1))) {qprobs <- c(qprobs, 1/num * i) } }

outvar <- rep(0, times=length(var))

outvar[is.na(var)] <- NA

cutpoints <- quantile(var,probs=qprobs, na.rm=TRUE)

for (k in (1:length(cutpoints))) { outvar[var>=cutpoints[k]] <- k; }

return(outvar)

}

removeNA<-function(i,j,m,wdf) {

vvv<-c(yvname[i],adjvv[[m]],subjvname,colvname,bvar,vname.start,timevar);

if (j<=nx) {vvv<-c(vvv,xvname[j]);} else {vvv<-c(vvv,xvname);}

vvv<-vvv[!is.na(vvv)]; vvv<-vvv[vvv>" "]

tmp<-is.na(wdf[,vvv]);

return(wdf[apply(tmp,1,sum)==0,])

}

if (!is.na(weights.var)) {weights<-WD[,weights.var];} else {weights<-1;}

WD<-cbind(WD,weights);

vlabelN<-(substr(vlabel,1,1)==" ");

vlabelZ<-vlabel[vlabelN];vlabelV<-vlabel[!vlabelN]

vnameV<-vname[!vlabelN];vnameZ<-vname[vlabelN]

w<-c("<!DOCTYPE html><html lang='zh'><head><meta charset='utf-8'></head><body>")

w<-c(w,paste("<h2>", title, "</h2>"))

if (length(avname)>0) {

if (sum((saf=="s" | saf=="S") & alv>0)>0) w<-c(w,"</br>Spline smoothing only applies for continuous variables")

if (!is.na(subjvname) & (sum((saf=="s" | saf=="S") & alv==0)>0)) w<-c(w,"</br>Generalized estimate equation could not be used with spline smoothing terms")

}

if (length(svname)>0) {

if (sum((sdf=="s" | sdf=="S") & slv>0)>0) w<-c(w,"</br>Spline smoothing only applies for continuous variables")

if (!is.na(subjvname) & (sum((sdf=="s" | sdf=="S") & slv==0)>0)) w<-c(w,"</br>Generalized estimate equation could not be used with spline smoothing terms")

}

allvname<-c(yvname,xvname,colvname,bvar,avname,svname,subjvname,vname.start,timevar,"weights");

allvname<-allvname[!is.na(allvname)]

WD<-WD[,allvname];

if (!is.na(subjvname)) WD<-WD[order(WD[,subjvname]),]

sxf<-as.numeric(sxf);sxf[is.na(sxf)]<-0;

if (sum(sxf>1 & xlv>0)>0) w<-c(w,"Categorizing only applies to continuous variables");

if (sum(sxf>1 & xlv==0)>0) {

t.xname<-NA;t.xlv<-NA; nx<-length(xvname)

for (i in 1:nx) {

if (sxf[i]>1 & xlv[i]==0) {

tmp.Xi<- rankvar(WD[,xvname[i]],sxf[i])

tmp.newcode <- tapply(WD[,xvname[i]],tmp.Xi,function(z) median(z,na.rm=TRUE))

tmp.low <- tapply(WD[,xvname[i]],tmp.Xi,function(z) min(z,na.rm=TRUE))

tmp.upp <- tapply(WD[,xvname[i]],tmp.Xi,function(z) max(z,na.rm=TRUE))

tmp.Xi2<- recodevar(tmp.Xi,(1:sxf[i])-1,tmp.newcode)

tmp.Xi<-cbind(tmp.Xi,tmp.Xi2)

tmp.NM<-paste(xvname[i],c("grp","grp.cont"),sep=".")

colnames(tmp.Xi)<-tmp.NM

WD<-cbind(WD,tmp.Xi)

t.xname<-c(t.xname,tmp.NM)

t.xlv<-c(t.xlv,sxf[i],0)

vnameV<-c(vnameV,tmp.NM)

vlabelV<-c(vlabelV,paste(vlabelV[vnameV==xvname[i]],c("group","group trend")))

vnameZ<-c(vnameZ,paste(tmp.NM[1],(1:sxf[i])-1,sep="."))

vlabelZ<-c(vlabelZ,paste(tmp.low,"-",tmp.upp))

} else {

t.xname<-c(t.xname,xvname[i]); t.xlv<-c(t.xlv,xlv[i])

}

}

xvname<-t.xname[-1]; xlv<-t.xlv[-1];

}

if (!is.na(subjvname)) {

if (length(avname)>0) saf<-rep(0,length(saf));

if (length(svname)>0) sdf<-rep(0,length(sdf));

WD<-WD[order(WD[,subjvname]),];

}

ny=length(yvname); nx=length(xvname);

xb<-vlabelV[match(xvname,vnameV)]; xb[is.na(xb)]<-xvname[is.na(xb)]

yb<-vlabelV[match(yvname,vnameV)]; yb[is.na(yb)]<-yvname[is.na(yb)]

xvname_ <- xvname

xvname_[xlv>0]<-paste("factor(",xvname[xlv>0],")",sep="")

xxname_<-list(NA); xxlbl_<-list(NA); xxlvl_<-list(NA)

for (j in (1:nx)) {

if (xlv[j]==0) {

xxname_[[j+1]]<-xvname[j];xxlbl_[[j+1]]<-xb[j];xxlvl_[[j+1]]<-0

} else {

xxlvl_[[j+1]]<-levels(factor(WD[,xvname[j]]))

tmp<-paste(xvname[j],".",xxlvl_[[j+1]],sep="")

xxlbl_[[j+1]]<-c(xb[j],vlabelZ[match(tmp,vnameZ)])

xxlbl_[[j+1]]<-paste(c("",rep("  ",length(xxlbl_[[j+1]])-1)),xxlbl_[[j+1]])

xxname_[[j+1]]<-c(xvname[j],paste("factor(",xvname[j],")",xxlvl_[[j+1]],sep=""))

}

}

xxname_<-xxname_[-1]; xxlbl_<-xxlbl_[-1]; xxlvl_<-xxlvl_[-1];

if (nx==1) par1<-1;

if (nx!=length(svname) & par1==4) par1<-1;

if (is.na(par1) | par1<1) par1<-1;

if (par1>1 & par1 < 4) {

tmp1<-xxname_[[1]]; tmp2<-xxlbl_[[1]]

for (j in 2:nx) {tmp1<-c(tmp1,xxname_[[j]]); tmp2<-c(tmp2,xxlbl_[[j]]);}

xxname_[[nx+1]]<-tmp1; xxlbl_[[nx+1]]<-tmp2;

xvname_<-c(xvname_,paste(xvname_,collapse="+"))

}

contx<-(sum(xlv>0)==0)

if (par1==3 & !is.na(bvar)) {w<-c(w,"</br>Column stratified variable was ignored"); bvar<-NA; bvname<-NA;}

fmlm<-" "; fmlb<-"Non-adjusted"; tmp<-""; adjvv<-list(NA); adjvb<-"None";

fmlm4<-list(rep("",nx))

fmlp<-ifelse(!is.na(subjvname), "gee", "glm");

na=0; avb=""; smoothav<-0; nadjm<-0

if (length(avname)>0) {

na<-length(avname)

avb<-vlabelV[match(avname,vnameV)];

avname_ <- avname

smoothavi<-((saf=="s" | saf=="S") & alv==0)

smoothav<-sum(smoothavi)

smoothavname<-avname[smoothavi]

avname_[smoothavi]<-paste("s(",avname[smoothavi],")",sep="")

avb1<-avb

avb1[smoothavi]<-paste(avb[smoothavi],"(Smooth)",sep="")

avname_[alv>0]<-paste("factor(",avname[alv>0],")",sep="")

fmlm<-c(fmlm,paste("+",paste(avname_,collapse="+")))

fmlb<-c(fmlb,"Adjust")

nadjm<-nadjm+1; tmp<-c(tmp,"I"); adjvv[[nadjm+1]]<-avname;

adjvb<-c(adjvb, paste(avb1, collapse="; "))

fmlp<-c(fmlp,ifelse(!is.na(subjvname), "gee", ifelse(smoothav>0, "gam", "glm")))

fmlm4<-c(fmlm4, list(rep("",nx)))

}

ns=0; svb=""; smoothsv<-0

if (length(svname)>0) {

svb<-vlabelV[match(svname,vnameV)];

svname_ <- svname

smoothsvi<-((sdf=="s" | sdf=="S") & slv==0)

smoothsv<-sum(smoothsvi)

smoothsvname<-svname[smoothsvi]

svname_[smoothsvi]<-paste("s(",svname[smoothsvi],")",sep="")

svb1<-svb

svb1[smoothsvi]<-paste(svb[smoothsvi],"(Smooth)",sep="")

svname_[slv>0]<-paste("factor(",svname[slv>0],")",sep="")

if (par1==4) {

fmlm<-c(fmlm, paste(fmlm[length(fmlm)],"+"));

fmlm4<-c(fmlm4, list(svname_));

} else {

fmlm<-c(fmlm,paste("+",paste(svname_,collapse="+")));

fmlm4<-c(fmlm4, list(rep("",nx)));

}

fmlb<-c(fmlb,"Adjust")

nadjm<-nadjm+1; tmp<-c(tmp,"II"); adjvv[[nadjm+1]]<-svname

adjvb2 <- paste(svb1, collapse="; ")

if (par1 == 4) {

if (avb>"") {adjvb2<-paste(avb1, "AND one in (", adjvb2,") respectively for each exposure X");

} else { adjvb2<-paste("ONE of (", adjvb2,") respectively for each exposure X");}

}

adjvb<-c(adjvb, adjvb2)

fmlp<-c(fmlp,ifelse(!is.na(subjvname), "gee", ifelse(smoothsv>0, "gam", "glm")))

}

if (chk==0 & length(fmlm)>1) {

fmlm<-fmlm[-1]; fmlb<-fmlb[-1]; tmp<-tmp[-1]; adjvv<-adjvv[-1]; adjvb<-adjvb[-1]; fmlp<-fmlp[-1]; fmlm4<-fmlm4[-1];

}

if (nadjm>1) fmlb<-paste(fmlb,tmp)

nmdl<-length(fmlm)

if (is.na(bvar) & !is.na(colvname) & nmdl==1 & par1!=3) {if ((ny==1) | (nx==1 & contx)) {bvar<-colvname; colvname<-NA;}}

if (is.na(colvname)) {

nclv<-1; clvb<-"Total"; clvb_<-"Total"

} else {

clv<-levels(factor(WD[,colvname])); nclv<-length(clv)+1

clvb_<-vlabelZ[match(paste(colvname,".",clv,sep=""),vnameZ)]; clvb_[is.na(clvb_)]<-clv[is.na(clvb_)];

clvb<-c(paste(vlabelV[vnameV==colvname],clvb_,sep="="),"Total");

clvb_<-c(clvb_,"Total")

WD<-WD[!is.na(WD[,colvname]),]

}

if (is.na(bvar)) {

blvb<-"Total"; blvb_<-"Total"

} else {

blv<-levels(factor(WD[,bvar])); nblv<-length(blv)+1

blvb_<-vlabelZ[match(paste(bvar,".",blv,sep=""),vnameZ)]; blvb_[is.na(blvb_)]<-blv[is.na(blvb_)];

blvb<-c(paste(vlabelV[vnameV==bvar],blvb_,sep="="),"Total");

blvb_<-c(blvb_,"Total")

WD<-WD[!is.na(WD[,bvar]),]

}

aa<-c(1,2,3,4)

for (i in 1:4) {

for (j in c(1:4)[-i]) {

for (k in c(1:4)[-c(i,j)]) aa<-rbind(aa,c(i,j,k,c(1:4)[-c(i,j,k)]))

}

}

colprn<-prn;

if (is.na(par3) | par3<1) par3<-1

rord<-aa[par3,]

if (!is.na(bvar)) {prn<-"S";

} else {

if (par3>1) {

rordc<-ifelse(rord[4]==1,3,4)

prn<-c("G","Y","M","X")[rord[rordc]]

if (prn=="X") {

if (!contx & nx>1) prn<-ifelse(nmdl>1, "M", ifelse(ny>nx & contx, "X", "Y"))

if (!contx & nx==1) prn<-"CX"

if (par1==2) {tmp<-ifelse(rordc==4,ifelse(rord[3]==1,2,3),2); prn<-c("G","Y","M","X")[rord[tmp]];}

}

} else {

prn<-ifelse(nmdl>1, "M", ifelse(ny>nx & contx, "X", "Y"))

if (par1==2) prn<-ifelse(nmdl>1, "M", "Y")

}

if (par1==3) prn<-"UM"

}

sink(paste(ofname,".lst",sep=""))

if (par1==2) {xbgn<-nx+1; xend<-nx+1;} else {xbgn<-1; xend<-nx;}

if (prn=="Y") {

tt<-c(0,0,0,0,"Exposure",yb); nn<-c(0,0,0,0,yb);

for (k in (1:nclv)) {

wdtmp0<-WD;

if (!is.na(colvname)) {

if (k<nclv) wdtmp0<-WD[WD[,colvname]==clv[k],];

print(paste("Stratified by",colvname, ":", clvb[k]))

}

for (m in 1:nmdl) {

for (j in (xbgn:xend)) {

colj<-cbind(k,0,m,j,xxlbl_[[j]])

nnj <-c(k,0,m,j)

for (i in (1:ny)) {

if (par1==4) {fml<-paste(yvname[i],"~",xvname_[j],fmlm[m],fmlm4[[m]][j]);} else {fml<-paste(yvname[i],"~",xvname_[j],fmlm[m]);}

wdtmp<-removeNA(i,j,m,wdtmp0)

if (!is.na(colvname)) {if (k==nclv) fml<-paste(fml,"+factor(",colvname,")",sep="");}

if (fmlp[m]=="gam") tmp.mdl<-setgam(fml,i)

if (fmlp[m]=="gee") tmp.mdl<-setgee(fml,i)

if (fmlp[m]=="glm") tmp.mdl<-setglm(fml,i)

tmpooi<-mdl2oo(tmp.mdl,xxname_[[j]],fmlp[m])

colj<-cbind(colj,tmpooi[[1]]); nnj<-c(nnj,tmpooi[[2]])

}

tt<-rbind(tt,colj); nn<-rbind(nn,nnj)

}

}

}

}

if (prn=="S") {

tt<-c(0,0,0,0,"Exposure",blvb); nn<-c(0,0,0,0,blvb);

for (k in (1:nclv)) {

wdtmp0<-WD;

if (!is.na(colvname)) {

if (k<nclv) wdtmp0<-WD[WD[,colvname]==clv[k],];

print(paste("Stratified by",colvname, ":", clvb[k]))

}

for (i in (1:ny)) {

for (m in 1:nmdl) {

for (j in (xbgn:xend)) {

colj<-cbind(k,i,m,j,xxlbl_[[j]]);

nnj <- c(k,i,m,j)

for (b in (1:nblv)) {

print(paste("Stratified by",bvar, ":", blvb[b]))

if (par1==4) {fml<-paste(yvname[i],"~",xvname_[j],fmlm[m],fmlm4[[m]][j]);} else {fml<-paste(yvname[i],"~",xvname_[j],fmlm[m]);}

if (b<nblv) {wdtmp1<-wdtmp0[wdtmp0[,bvar]==blv[b],];} else {wdtmp1<-wdtmp0; fml<-paste(fml,"+factor(",bvar,")",sep="");}

wdtmp<-removeNA(i,j,m,wdtmp1)

if (!is.na(colvname)) {if (k==nclv) fml<-paste(fml,"+factor(",colvname,")",sep="");}

if (fmlp[m]=="gam") tmp.mdl<-setgam(fml,i)

if (fmlp[m]=="gee") tmp.mdl<-setgee(fml,i)

if (fmlp[m]=="glm") tmp.mdl<-setglm(fml,i)

tmpooi<-mdl2oo(tmp.mdl,xxname_[[j]],fmlp[m])

colj<-cbind(colj,tmpooi[[1]]); nnj<-c(nnj,tmpooi[[2]])

}

tt<-rbind(tt,colj); nn<-rbind(nn,nnj)

}

}

}

}

}

if (prn=="M") {

tt<-c(0,0,0,0,"Exposure",fmlb); nn<-c(0,0,0,0,fmlb)

for (k in (1:nclv)) {

wdtmp0<-WD;

if (!is.na(colvname)) {

if (k<nclv) wdtmp0<-WD[WD[,colvname]==clv[k],];

print(paste("Stratified by",colvname, ":", clvb[k]))

}

for (i in 1:ny) {

for (j in xbgn:xend) {

colj<-cbind(k,i,0,j,xxlbl_[[j]]); nnj<-c(k,i,0,j)

for (m in 1:nmdl) {

if (par1==4) {fml<-paste(yvname[i],"~",xvname_[j],fmlm[m],fmlm4[[m]][j]);} else {fml<-paste(yvname[i],"~",xvname_[j],fmlm[m]);}

wdtmp<-removeNA(i,j,m,wdtmp0)

if (!is.na(colvname)) {if (k==nclv) fml<-paste(fml,"+factor(",colvname,")",sep="");}

if (fmlp[m]=="gam") tmp.mdl<-setgam(fml,i)

if (fmlp[m]=="gee") tmp.mdl<-setgee(fml,i)

if (fmlp[m]=="glm") tmp.mdl<-setglm(fml,i)

tmpooi<-mdl2oo(tmp.mdl,xxname_[[j]],fmlp[m])

colj<-cbind(colj,tmpooi[[1]]); nnj<-c(nnj,tmpooi[[2]])

}

tt<-rbind(tt,colj); nn<-rbind(nn,nnj)

}

}

}

}

if (prn=="X") {

tt<-c(0,0,0,0,"Outcome",xb); nn<-c(0,0,0,0,xb);

for (k in (1:nclv)) {

wdtmp0<-WD;

if (!is.na(colvname)) {

if (k<nclv) wdtmp0<-WD[WD[,colvname]==clv[k],];

print(paste("Stratified by",colvname, ":", clvb[k]))

}

for (m in 1:nmdl) {

for (i in (1:ny)) {

colj<-cbind(k,i,m,0,yb[i])

nnj <-c(k,i,m,0)

for (j in (1:nx)) {

if (par1==4) {fml<-paste(yvname[i],"~",xvname_[j],fmlm[m],fmlm4[[m]][j]);} else {fml<-paste(yvname[i],"~",xvname_[j],fmlm[m]);}

wdtmp<-removeNA(i,j,m,wdtmp0)

if (!is.na(colvname)) {if (k==nclv) fml<-paste(fml,"+factor(",colvname,")",sep="");}

if (fmlp[m]=="gam") tmp.mdl<-setgam(fml,i)

if (fmlp[m]=="gee") tmp.mdl<-setgee(fml,i)

if (fmlp[m]=="glm") tmp.mdl<-setglm(fml,i)

tmpooi<-mdl2oo(tmp.mdl,xxname_[[j]],fmlp[m])

colj<-cbind(colj,tmpooi[[1]]); nnj<-c(nnj,tmpooi[[2]])

}

tt<-rbind(tt,colj); nn<-rbind(nn,nnj)

}

}

}

}

if (prn=="CX") {

tt<-c(0,0,0,0,"Outcome",xxlbl_[[1]][-1]); nn<-c(0,0,0,0,xb[1]);

nxl<-length(xxlbl_[[1]])-1

for (k in (1:nclv)) {

wdtmp0<-WD;

if (!is.na(colvname)) {

if (k<nclv) wdtmp0<-WD[WD[,colvname]==clv[k],];

print(paste("Stratified by",colvname, ":", clvb[k]))

}

for (m in 1:nmdl) {

for (i in (1:ny)) {

colj<-c(k,i,m,0,yb[i])

nnj <-c(k,i,m,0)

fml<-paste(yvname[i],"~",xvname_[1],fmlm[m]);

wdtmp<-removeNA(i,1,m,wdtmp0)

if (!is.na(colvname)) {if (k==nclv) fml<-paste(fml,"+factor(",colvname,")",sep="");}

if (fmlp[m]=="gam") tmp.mdl<-setgam(fml,i)

if (fmlp[m]=="gee") tmp.mdl<-setgee(fml,i)

if (fmlp[m]=="glm") tmp.mdl<-setglm(fml,i)

tmpooi<-mdl2oo(tmp.mdl,xxname_[[1]],fmlp[m])

colj<-c(colj,tmpooi[[1]][-1]); nnj<-c(nnj,tmpooi[[2]])

tt<-rbind(tt,colj); nn<-rbind(nn,nnj)

}

}

}

}

if (prn=="UM") {

tt<-c(0,0,0,0,"Exposure","Univariable","Multivariable"); nn<-c(0,0,0,0,"Univariable","Multivariable")

for (k in (1:nclv)) {

wdtmp0<-WD;

if (!is.na(colvname)) {

if (k<nclv) wdtmp0<-WD[WD[,colvname]==clv[k],];

print(paste("Stratified by",colvname, ":", clvb[k]))

}

for (i in 1:ny) {

for (m in 1:nmdl) {

colm<-rep(NA,6); nnm<-rep(NA,5)

for (j in 1:(nx+1)) {

colj<-cbind(k,i,m,j,xxlbl_[[j]]); nnj<-c(k,i,m,j)

if (par1==4) {fml<-paste(yvname[i],"~",xvname_[j],fmlm[m],fmlm4[[m]][j]);} else {fml<-paste(yvname[i],"~",xvname_[j],fmlm[m]);}

wdtmp<-removeNA(i,j,m,wdtmp0)

if (!is.na(colvname)) {if (k==nclv) fml<-paste(fml,"+factor(",colvname,")",sep="");}

if (fmlp[m]=="gam") tmp.mdl<-setgam(fml,i)

if (fmlp[m]=="gee") tmp.mdl<-setgee(fml,i)

if (fmlp[m]=="glm") tmp.mdl<-setglm(fml,i)

tmpooi<-mdl2oo(tmp.mdl,xxname_[[j]],fmlp[m])

colj<-cbind(colj,tmpooi[[1]]); nnj<-c(nnj,tmpooi[[2]])

if (j<=nx) {colm<-rbind(colm,colj); nnm<-rbind(nnm,nnj);

} else {colm<-cbind(colm[-1,],tmpooi[[1]]); nnm<-cbind(nnm[-1,],tmpooi[[2]]);}

}

tt<-rbind(tt,colm); nn<-rbind(nn,nnm)

}

}

}

}

sink()

if (!contx & prn!="CX") rord<-c(rord[rord!=4],4)

if (prn=="X" | prn=="CX") rord<-rord[rord!=4]

if (prn=="Y") rord<-rord[rord!=2]

if (prn=="M") rord<-rord[rord!=3]

if (nx==1 & contx) rord<-rord[rord!=4]

if (nmdl==1) rord<-rord[rord!=3]

if (ny==1) rord<-rord[rord!=2]

if (is.na(colvname)) rord<-rord[rord!=1]

if (length(rord)==0) rord<-1

nrr<-length(rord)

for (i in nrr:1) {nn<-nn[order(as.numeric(nn[,rord[i]])),];tt<-tt[order(as.numeric(tt[,rord[i]])),];}

if (nn[2,4]>0) {nn[,4]<-c("Exposure",xb[as.numeric(nn[-1,4])]); } else {nn<-nn[,-4];}

if (nn[2,3]>0) {nn[,3]<-c("Model",fmlb[as.numeric(nn[-1,3])]); } else {nn<-nn[,-3];}

if (nn[2,2]>0) {nn[,2]<-c("Outcome",yb[as.numeric(nn[-1,2])]); } else {nn<-nn[,-2];}

if (!is.na(colvname)) {nn[,1]<-c(vlabel[vname==colvname],clvb_[as.numeric(nn[-1,1])]);} else {nn<-nn[,-1];}

tb<-matrix(as.numeric(tt[,c(1:4)]),ncol=4);

if (!is.na(colvname)) {tt[,1]<-c(vlabelV[vnameV==colvname],clvb[tb[-1,1]]);}

if (ny>1) {tt[,2]<-c("Outcome",yb[tb[-1,2]]);}

if (nmdl>1) {tt[,3]<-c("Model",fmlb[tb[-1,3]]);}

nrr1<-nrr-1; oo<-tt[1,]; nc<-ncol(tt)-5; nr<-nrow(tt)

for (i in 2:nr) {

if (nrr>1) {

for (j in 1:nrr1) {

if (tb[i,rord[j]]!=tb[i-1,rord[j]]) oo<-rbind(oo,c(rep(tt[i,rord[j]],5),rep(" ",nc)))

}

}

oo<-rbind(oo,tt[i,])

}

if (rord[nrr]!=4 & tt[1,rord[nrr]]!="0") {oo<-cbind(oo[,rord[nrr]],oo[,-(1:5)]);} else {oo<-oo[,-(1:4)]}

w<-c(w,"</br><table border=3>", mat2htmltable(oo), "</table>")

prnopt<-c("β (95%CI) Pvalue / OR (95%CI) Pvalue", "β (95%CI) Pvalue", "β+se / OR (95%CI) *P<0.05 **P<0.01 ***P<0.001")
